# Engineering the oleaginous yeast *Yarrowia lipolytica* for high-level resveratrol production

**DOI:** 10.1016/j.ymben.2020.08.009

**Published:** 2020-11

**Authors:** Javier Sáez-Sáez, Guokun Wang, Eko Roy Marella, Suresh Sudarsan, Marc Cernuda Pastor, Irina Borodina

**Affiliations:** The Novo Nordisk Foundation Center for Biosustainability, Technical University of Denmark, DK-2800, Kgs. Lyngby, Denmark

**Keywords:** Aromatics, Shikimate pathway, *p*-coumaric acid, Phenylpropanoids

## Abstract

Resveratrol is a plant secondary metabolite with multiple health-beneficial properties. Microbial production of resveratrol in model microorganisms requires extensive engineering to reach commercially viable levels. Here, we explored the potential of the non-conventional yeast *Yarrowia lipolytica* to produce resveratrol and several other shikimate pathway-derived metabolites (*p*-coumaric acid, *cis*,*cis*-muconic acid, and salicylic acid). The *Y. lipolytica* strain expressing a heterologous pathway produced 52.1 ± 1.2 mg/L resveratrol in a small-scale cultivation. The titer increased to 409.0 ± 1.2 mg/L when the strain was further engineered with feedback-insensitive alleles of the key genes in the shikimate pathway and with five additional copies of the heterologous biosynthetic genes. In controlled fed-batch bioreactor, the strain produced 12.4 ± 0.3 g/L resveratrol, the highest reported titer to date for *de novo* resveratrol production, with a yield on glucose of 54.4 ± 1.6 mg/g and a productivity of 0.14 ± 0.01 g/L/h. The study showed that *Y. lipolytica* is an attractive host organism for the production of resveratrol and possibly other shikimate-pathway derived metabolites.

## Highlights

• Oleaginous yeast *Y. lipolytica* was engineered for production of aromatic compounds.
• High resveratrol production required increased activities of Aro4p and Aro7p.
• Multiple integration of resveratrol biosynthetic genes improved production.
• Fed-batch fermentation enabled *de novo* production of 12.4 g/L resveratrol.

## 1 Introduction

Resveratrol (3,5,4′-trihydroxy-*trans*-stilbene) is a stilbenoid naturally present in grapes, berries, Japanese knotweed, and peanuts (Burns et al., 2002). In plants, it serves as phytoalexin, produced in defense against injuries and microbial infections (Langcake and Pryce, 1976; Vestergaard and Ingmer, 2019). In the past two decades, over 1500 studies have been published on the health effects of resveratrol (PubMed, 2020). Clinical trials have proven that resveratrol beneficially influences disease biomarkers of diabetes, cardiovascular diseases, and neurological disorders (Bhatt et al., 2012; Brasnyó et al., 2011; Tomé-Carneiro et al., 2012; Turner et al., 2015). Resveratrol is commercialized as food and cosmetics ingredient and is sold as food supplement. The current market is around $97.7 million and is expected to grow at a compound annual growth rate (CAGR) of 8.1% from 2018 to 2028 (Future Market Insights, 2019). Extraction from Japanese knotweed remains the main source of resveratrol, but chemical synthesis by DSM and microbial fermentation by Evolva are gaining ground in the market (DSM, 2014; Evolva, 2019; Future Market Insights, 2019). Microbial fermentation process of resveratrol production has a number of advantages, such as low cost, high purity of the product, and independence on season.

Resveratrol is biosynthesized in plants via the phenylpropanoids pathway, using as precursors the aromatic amino acids L-phenylananine (L-Phe) or L-tyrosine (L-Tyr) (Sparvoli et al., 1994; Rosler et al., 1997). The first biosynthetic step involves the deamination of L-Phe/L-Tyr by phenylalanine/tyrosine ammonia lyase (PAL/TAL). In the L-Phe branch, PAL activity yields *trans*-cinnamic acid, which is hydroxylated into *p*-coumaric acid by cinnamic acid hydroxylase (C4H), a cytochrome P450 enzyme (Winkel-Shirley, 2001). Alternatively, TAL activity in the L-Tyr branch results in *p*-coumaric acid in a single enzymatic step (Jendresen et al., 2015). Further, coenzyme A is attached to *p*-coumaric acid by 4-coumaroyl-CoA ligase (4CL), resulting in 4-coumaroyl-CoA. In a final step, resveratrol synthase, a type III polyketide synthase (PKS), incorporates three units of malonyl-CoA to synthesize resveratrol (Winkel-Shirley, 2001). Microbial production of resveratrol by heterologous expression of the plant pathway has been successfully carried out in several hosts (Table 1, Supplementary Table S1) (Thapa et al., 2019). Extensive metabolic engineering strategies have also been implemented to improve resveratrol titers, mainly aiming at rewiring carbon metabolism towards shikimate pathway, increasing L-Tyr/L-Phe and malonyl-CoA precursor supply, alleviating feedback regulations, or tuning gene copy number in the heterologous pathway (Liu et al., 2019; Li et al., 2015, 2016; Lim et al., 2011). Despite the efforts, the *de novo* production levels reported in scientific publications are still low, with the highest titer of 0.8 g/L achieved in *S. cerevisiae* (Li et al., 2016). Gram per liter titers have only been reached in *E. coli* by co-feeding *p*-coumaric acid (Lim et al., 2011), however addition of *p*-coumaric acid is not an option for a large-scale fermentation.

**Table 1:** **Resveratrol production in microbial hosts**. The table includes the top producing strains per microorganism, considering also cultivations in which pathway precursors were fed. *Cerulenin was supplemented to the cultivation. **Refer to publication for detailed list of genetic modifications. n/a: metric not available as cultivation time was not reported. 4CL: 4-coumaroyl-CoA, *STS*: stilbene synthase, *PAL*: phenylalanine ammonia-lyase, *C4H*: cinnamate-4-hydroxylase, *VST*: resveratrol synthase, *ACS*: acetyl-CoA synthase, *ATR2*: cytochrome P450 reductase, *ARO4*: 3-deoxy-D-arabinoheptulosonate-7-phosphate (DAHP) synthase, *ARO7*: chorismate mutase, *CYB5*: cytochrome *b*5, *ACC1*: acetyl-CoA carboxylase 1, *ARO10*: transaminated amino acid decarboxylase, *PEX10*: peroxisomal biogenesis factor 10, *TAL*: tyrosine ammonia-lyase, *pks*: polyketide synthase, fbr: feedback-resistant.

Microbial host	Genetic modifications	Metrics	Substrate/Precursor fed	Reference
*E. coli*	4CL (*A. thaliana*) *STS* (*V. vinifera*)	Titer: 2.3 g/L Yield: 1.01 g/g *p*-coumaric acid Productivity: 95.83 mg/L/h	Glycerol/*p*-coumaric acid*	Lim et al. (2011)
*S. cerevisiae*	*PAL* (*A. thaliana*) *C4H* (*A. thaliana*) 4CL (*A. thaliana*) *VST* (*V. vinifera*) *ACS* (*S. enterica*) *ATR2* (*A. thaliana*) Overexpression of *ARO4*^fbr^, *ARO7*^fbr^, *CYB5,* and *ACC1* *ΔARO10*	Titer: 812 mg/L Yield: 8.87 mg/g glucose Productivity: 7.38 mg/L/h	Glucose/none	Li et al. (2016)
*Y. lipolytica*	4CL (*N. tabacum*) *STS* (*A. hypogaea*) Overexpression of *PEX10* and *ACC1*	Titer: 48.7 mg/L Yield: 0.15 g/g *p*-coumaric acid Productivity: 0.29 mg/L/h	Glucose/*p*-coumaric acid	Palmer et al. (2020)
*C. glutamicum*	*STS* (*A. hypogaea*) 4CL (*P. crispum*) **	Titer: 158 mg/L Yield: 0.19 g/g *p*-coumaric acid Productivity: 2.19 mg/L/h	Glucose/*p*-coumaric acid*	Kallscheuer et al. (2016)
*L. lactis*	*TAL,* 4CL*, STS, ACC* (different sources)	Titer: 1.27 mg/L Yield: 0.13 mg/g glucose Productivity: n/a	Glucose/none	Gaspar et al. (2016)
*S. venezuelae*	*STS* (*A. hypogaea*) 4CL (*S. coelicolor*) Pikromycin *pks* deletion	Titer: 0.4 mg/L Yield: 0.002 g/g *p*-coumaric acid Productivity: 0.006 mg/L/h	Sucrose/*p*-coumaric acid	Park et al. (2009)

*Yarrowia lipolytica* is an emerging industrial host for the production of lipids, polyunsaturated fatty acids, and organic acids. As an oleaginous yeast, *Y. lipolytica* is naturally endowed with high fluxes towards malonyl-CoA and the pentose phosphate pathway (PPP) (Wasylenko et al., 2015; Blank et al., 2005). These metabolic traits could be particularly relevant for the production of shikimate pathway-derived compounds and plant natural products requiring aromatic amino acids and malonyl-CoA-derived building blocks, such as phenylpropanoids. Indeed, this yeast was recently shown as an attractive host for the biosynthesis of several plant flavonoids, including naringenin, eriodictyol, taxifolin, and other aromatic compounds (Lv et al., 2019, 2020; Palmer et al., 2020; Gu et al., 2020a, 2020b; Shang et al., 2020; Wei et al., 2020). Resveratrol in particular has also been produced in *Y. lipolytica*, first described by DuPont and more recently in studies exploring the ability of the non-conventional yeast as production chassis for different polyketides and aromatics (Supplementary Table S1) (Huang et al., 2006; Palmer et al., 2020; Gu et al., 2020a).

In this work, we have investigated the potential of *Y. lipolytica* for the production of several shikimate pathway-derived metabolites: *p*-coumaric acid, resveratrol, *cis*,*cis*-muconic acid, and salicylic acid.

## 2 Materials and methods

### 2.1 Strain construction and cultivation

All *Y. lipolytica* strains constructed in this study are derived from ST6512, a W29 (NRRL Y-63746) strain harboring Cas9 in *KU70 locus* (Marella et al., 2019). The complete list of strains, plasmids, biobricks, and primers used in this work are available in the supplementary information 2 (Supplementary Tables S2–S5). The strains are available upon request. The strain ST9671 has been deposited with Euroscarf collection (accession number Y41418). Unless otherwise stated, for pre-culture, strain construction, propagation, and cryostocking, yeast strains were grown at 30 °C and 250 rpm (Thermo Fisher Scientific MaxQ8000) in YPD medium (10 g/L yeast extract, 20 g/L peptone, 20 g/L D-glucose). Media for plates contained 20 g/L agar.

Plasmids required for genome engineering were constructed using the set of vectors from EasyCloneYALI as backbones (Holkenbrink et al., 2018). Integrative vector backbones were PCR-amplified with compatible USER overhangs, while guide-RNA (gRNA) vector backbones for gene deletion were linearized by digestion. Synthetic genes required for *p*-coumaric acid, resveratrol, *cis*,*cis*-muconic acid and salicylic acid production were codon-optimized for *Y. lipolytica* using Thermo Fisher Scientific webtool and ordered as GeneArt Strings DNA Fragments. The DNA sequence of the synthetic genes is available in the supplementary information 2 (Supplementary Table S6). Plasmid assembly and cloning was performed according to EasyCloneYALI instructions (Holkenbrink et al., 2018). For gene deletions, DNA fragments consisting of 400–600 bp up- and downstream of the target gene were used as repair template (800–1200 bp total). All constructed plasmids were verified by Sanger sequencing (Eurofins Scientific SE). Yeast transformations were performed using a lithium acetate-based protocol as previously described (Holkenbrink et al., 2018). Transformants were selected using natMX or hphMX resistance markers. YPD plates for natMX or hphMX selection contained 250 mg/L of nourseothricin (Jena Bioscience, AB-101) or 400 mg/L hygromycin B (Invitrogen, 10687010), respectively.

### 2.2 Small-scale production and degradation assays

Standard production and degradation assays were performed in mineral medium containing 20 g/L D-glucose as carbon source (pH 6.0, adjusted with KOH), as described in (Jensen et al., 2014).

Strains from cryostocks were streaked onto YPD plates and incubated for 48 h at 30 °C. Three single colonies from the YPD plate were inoculated for pre-culture in 24 deep-well plates (Enzyscreen B.V., CR1424) containing 3 mL of mineral medium, and incubated at 30 °C for 48 h and 300 rpm agitation at 5 cm orbit cast, reaching the stationary phase. An adequate volume of pre-culture to start with an initial optical density (OD_600_) of 0.1 was transferred to a fresh 24 deep-well plate containing 3 mL of medium, which was incubated for 72 h under the same conditions, reaching the stationary phase. After 72 h of cultivation, OD_600_ measurements and samples for HPLC analysis were prepared according to the procedure described in section 2.4.

In the initial screening of *p*-coumaric acid and resveratrol production, the medium was supplemented with 2 mM L-tyrosine. The degradation assays were performed following the same conditions, but supplementing the mineral medium in the 72 h cultivation with the target compound at different concentrations.

Medium optimization for resveratrol production was carried out using mineral medium, YNB and YP with either 20/80 g/L glucose or 20/80 g/L glycerol as carbon source. YP contained 10 g/L yeast extract, 20 g/L peptone; YNB contained 6.7 g/L Yeast Nitrogen Base Without Amino Acids (Sigma-Aldrich, Y0626) and was adjusted to pH 6.0 with KOH. Pre-culture was carried out in standard YPD for 48 h in 24 deep-well plates. Pre-cultivation samples were centrifuged for 5 min at 3000×*g*, washed twice with sterile Milli-Q® water. An adequate volume to start at an initial OD_600_ of 0.1 was used to inoculate each of the cultivations in a new 24 deep-well plate, which was incubated for 96 h and sampled every 24 h. In the assay to assess the effect of antifoam on resveratrol production, antifoam 204 (Sigma-Aldrich, A6426) was added to the standard mineral medium with 20 g/L glucose at concentrations of 1%, 3% and 5% v/v.

### 2.3 Fed-batch fermentation in bioreactor

For seed culture preparation, strain from cryostock was streaked onto a YPD plate and grown at 30 °C for 48 h. Biomass from the plate was transferred to 1 mL YPD in a 14 mL pre-culture tube and incubated at 30 °C for 18 h. A pre-culture volume of 250 μL was used to inoculate 50 mL of YPD in a 250 mL baffled shake flask and incubated at 30 °C for 24 h and 250 rpm. The content of the shake flask was centrifuged for 5 min at 5000×*g*, washed twice with mineral medium and concentrated in 5 mL volume. This cell suspension was used to inoculate the reactors to an initial OD_600_ of 1.0.

The fermentation was carried out in duplicate in 1 L bioreactors (BIOSTAT® Q plus, Sartorius, Goettingen, Germany) equipped with measurement probes for pH, dissolved oxygen (DO) and temperature. Aeration was achieved with a horseshoe sparger. Off-gas O_2_ and CO_2_ levels were logged with a Prima BT MS (Thermo Fisher Scientific). Fermentation was carried out at 30 °C and pH was maintained at 6.0 by automatic addition of 5 M KOH. The reactors initially contained 400 mL of mineral medium. One liter of medium contained 5 g (NH_4_)_2_SO_4_, 3 g KH_2_PO_4_, 0.5 g MgSO_4_·7H_2_O, 40 g D-glucose, 2 mL trace metal solution, 1 mL vitamin solution, and 0.4 mL antifoam 204. The trace metal and vitamin solutions were prepared as in (Jensen et al., 2014). Stirring and aeration rate were initiated at 540 rpm and 0.5 standard liter per minute (SLPM), respectively. The feed addition started when DO decreased from the initial 100% to 35%. During the fed-batch phase, DO was maintained at 20% by a two-level cascade of stirring (between 540 and 1200 rpm) and airflow (between 0.5 and 1.5 SLPM). The initial feed rate was 1.0 g/h, with an exponential increase of 0.05 h^−1^. When stirring and airflow reached maximum setpoints, feeding was changed to a constant feed rate until the end of the fermentation. The feed medium contained 460 g/L of D-glucose and 0.6 mL/L of antifoam, while the rest of the medium components were the same as in batch mineral medium, but at 10-fold higher concentration. Additional sterile antifoam 204 was manually added to the reactors when foaming was observed. Sampling was carried out three times per day to measure OD_600_, cell dry weight, and resveratrol.

### 2.4 Analytical methods

OD_600_ measurements were performed in a NanoPhotometer® P-Class (Implen GmbH) with 1.5 mL volume and 1 cm path cuvettes (Brand GmbH). The cell dry weight measurements were performed using Whatman™ cellulose nitrate membrane filters disc with 0.45 μm pore size (VWR, 7184–004). 500 μL of reactor sample was centrifuged at 17,000×*g* for 5 min. Cell pellet was resuspended in 1 mL deionized water and the suspension was loaded onto the filter placed on a filtration unit. Filters were washed with 5 mL deionized water through vacuum. Before and after loading the sample, filters were dried using a microwave and weighed, as previously described (Marella et al., 2019). Cell dry weight was calculated as the difference in filter weights before and after loading the cell suspension. Measurements were performed in duplicate for each of the reactors.

For salicylic acid and *cis*,*cis*-muconic acid quantification, cultivation samples were centrifuged at 17,000×*g* for 5 min and the supernatant used for analysis. In the case of *p*-coumaric acid and resveratrol, cultivation samples were mixed with an equal volume of absolute ethanol, vortexed thoroughly for 10 s, and centrifuged at 17,000×*g* for 5 min. Supernatants were used for analysis. In the case of intracellular analysis of resveratrol, 1 mL of broth was centrifuged at 17,000×*g* for 5 min and the cell pellet was washed twice with 1 mL 50% v/v ethanol. Cells were resuspended in 500 μL of water and transferred to 2 mL screwcap tubes filled with 300 μL of glass beads (212–300 μm particle size, Sigma-Aldrich, G1277). Cell suspensions were disrupted with Precellys® 24 (Bertin Instruments) for five cycles of 20 s shaking at 5000 rpm with 5-min pauses between cycles, when the samples were placed on ice. 500 μL of absolute ethanol was added to the disrupted cells and vortexed thoroughly. Samples were centrifuged at 17,000×*g* for 5 min and the supernatant was used for the analysis. The samples from small-scale cultivations were analyzed on HPLC the same day. Bioreactor samples were prepared for HPLC and stored at −20 °C until analysis.

Quantification of all compounds was performed with Dionex UltiMate 3000 HPLC (Thermo Fisher Scientific), equipped with a DAD-3000 UV/Vis detector (Dionex) and a RI-101 Refractive Index Detector (Dionex). HPLC-grade solvents were used for the mobile phase. Peaks corresponding to the target compounds were identified by comparison to prepared standards (Sigma-Aldrich). Peak areas were used for compound quantification using external standard calibration method. Analysis of HPLC results was performed using the software Chromeleon 7 (ThermoFisher Scientific).

For resveratrol and *p*-coumaric acid, the HPLC system was equipped with a Discovery HS F5 150 mm × 2.1 mm column, particle size 3 μm (Supelco, 567503-U). The column oven temperature was set at 30 °C and the flow rate to 0.7 mL/min 5 μL of sample was injected for the quantification. Solvent A was 10 mM ammonium formate (pH 3.0, adjusted by formic acid). Solvent B was acetonitrile. Solvent composition was initially A = 95.0%, and B = 5.0%, which was kept until 0.5 min. Then, solvent composition was changed following a linear gradient until A = 40.0%, and B = 60.0% at 7.0 min. These conditions were kept constant for 2.5 min (7.0–9.5 min). The solvent composition was returned linearly to the initial conditions (A = 95.0%, B = 5.0%) at 9.6 min, and remained unchanged until the end of the run (9.6-12 min). *p*-Coumaric acid was detected at a retention time of 4.7 min, using the absorbance at 277 nm for the quantification. Resveratrol was detected at a retention time of 5.7 min, using the absorbance at 333 nm for the quantification.

For salicylic acid, the HPLC system was equipped with a Cortecs UPLC T3 2.1 × 150 mm column, particle size 1.6 μm, pore size 120 Å (Waters, 186008500). The column oven temperature was set at 30 °C and the flow rate to 0.3 mL/min. Solvent A was 0.1% formic acid in Milli-Q® water; solvent B was acetonitrile. 5 μL of sample was injected for the quantification. The initial solvent composition was A = 90.0%, and B = 10.0%, which was kept until 0.5 min. Solvent composition was then changed following a linear gradient until % A = 5.0 and % B = 95.0 at 7 min. This solvent composition was returned linearly to the initial conditions (A = 90.0%, B = 10.0%) at 7.1 min, and remained unchanged until the end of the run (7.1-10 min). Salicylic acid was detected at a retention time of 6.6 min, using the absorbance at 250 nm for the quantification.

Quantification of *cis*,*cis*-muconic acid and pathway intermediates was performed using previously described methods (Wang et al., 2020).

Quantification of glucose, glycerol and organic acids was carried out on Aminex HPX-87H column (Bio-Rad Laboratories, USA) as described before (Borja et al., 2019), but with a column temperature of 60 °C.

## 3 Results

### *3.1* *Yarrowia lipolytica* was readily able to produce aromatics

**Fig. 1:**
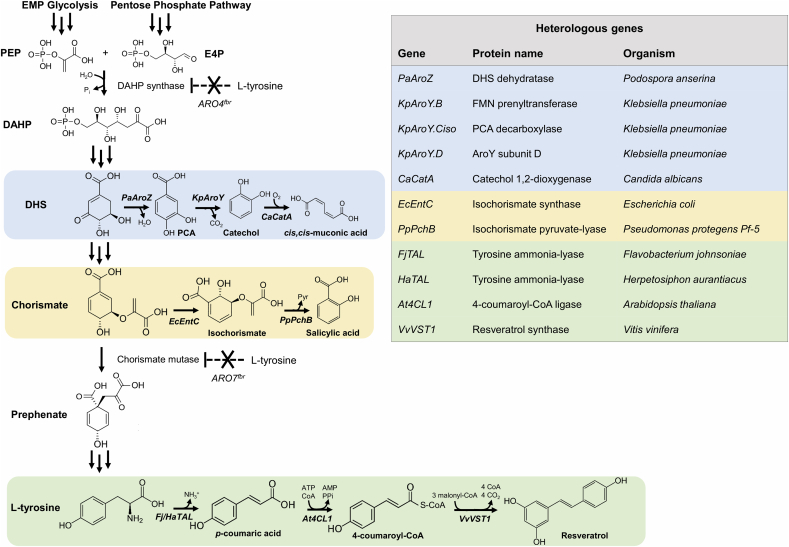
**Biosynthetic pathways towards different aromatic compounds in engineered *Y. lipolytica***. The different aromatic metabolites are synthesized using either intermediates from the shikimate pathway or the aromatic amino acid L-tyrosine. Different colors show the different heterologous pathways. Multiple arrows represent multiple enzymatic steps, dashed arrows indicate feedback-regulated steps. PEP: phosphoenolpyruvate E4P: erythrose 4-phosphate, DAHP: 3-deoxy-D-arabinoheptulosonate 7-phosphate, DHS: 3-dehydroshikimate. PCA: protocatechuic acid, FMN: flavin mononucleotide. *ARO4*: DAHP synthase, *ARO7*: chorismate mutase, fbr: feedback-resistant. (For interpretation of the references to color in this figure legend, the reader is referred to the Web version of this article.)

Insufficient intracellular levels of malonyl-CoA are a known limitation for the production of phenylpropanoids (Johnson et al., 2017). As the native metabolic traits of *Y. lipolytica* overcome this limitation, we tested its potential to synthesize the stilbenoid resveratrol. Additionally, we also evaluated its ability to produce *cis*,*cis*-muconic acid and salicylic acid, compounds that do not require malonyl-CoA building blocks and branch off from different precursors upstream in the shikimate pathway (Fig. 1).

**Fig. 2:**
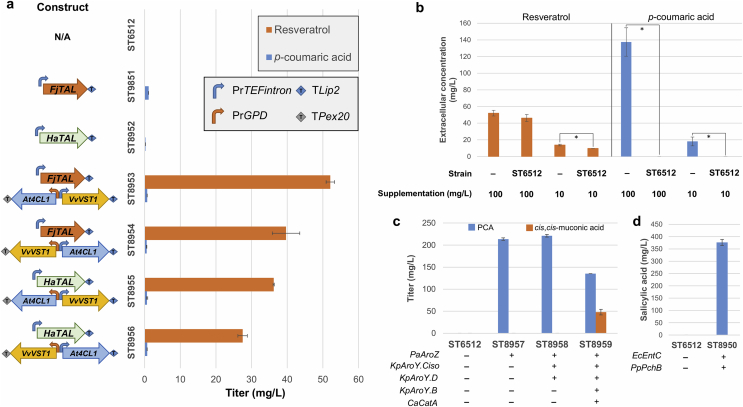
**Titers of *p*-coumaric acid, resveratrol, *cis*,*cis*-muconic acid and salicylic in engineered *Y. lipolytica* strains.** Cultivations were carried out for 72 h in 24 deep-well plates containing mineral medium with 20 g/L glucose. Extracellular content was subjected to HPLC analysis. Error bars represent standard deviation from at least three biological replicates. “-” and “+” symbols indicate absence or presence of the corresponding genetic modification, respectively. a) Production of *p*-coumaric acid and resveratrol with supplementation of 2 mM L-tyrosine. b) Degradation assays for *p*-coumaric acid and resveratrol in strain ST6512, at different supplementation concentrations. “-” represents a medium control, non-inoculated. c) Production of *cis*,*cis*-muconic acid and the pathway intermediate protocatechuic acid (PCA). d) Production of salicylic acid. Statistical analysis was performed using Student's t-test (two-tailed; *P ≤ 0.05 two-sample unequal variance).

For the biosynthesis of resveratrol, we first tested two different TAL enzymes from *Herpetosiphon aurantiacus* (HaTAL) and *Flavobacterium johnsoniae* (FjTAL) producing the intermediate *p*-coumaric acid that have been shown to perform best in *S. cerevisiae* (Jendresen et al., 2015). The pathway was further extended up to resveratrol with *At4CL1* from *Arabidopsis thaliana* and *VvVST1* from *Vitis vinifera*, genes that were also successfully expressed in *S. cerevisiae* (Li et al., 2015). In a similar fashion to Li et al. work, we used different combinations of promoters with strong and constitutive expression like *GPD* and *TEFintron* to assess the best performing strain, using mineral medium with 2 mM L-tyrosine for the cultivation (Li et al., 2015; Holkenbrink et al., 2018). We evaluated the performance of the genes and combinations of promoters both in terms of titer and specific yield (Fig. 2a, Supplementary Fig. S1a).

The best production of *p*-coumaric acid was achieved in strain ST8951 harboring FjTAL, but the titer remained low (1.11 ± 0.01 mg/L). ST8952 containing HaTAL produced 0.21 ± 0.01 mg/L *p*-coumaric acid (Fig. 2a, Supplementary Fig. S1a). Based on these results and due to the native ability of *Y. lipolytica* to utilize a wide range of substrates (Abdel-Mawgoud et al., 2018), we hypothesized that *p*-coumaric acid could be consumed. In order to evaluate this, we performed a degradation assay with the parental strain ST6512 by supplementing *p*-coumaric acid to mineral medium. After 72 h, no *p*-coumaric acid was detected in the medium, suggesting its complete consumption by *Y. lipolytica* (Fig. 2b). This was not observed in the degradation assays for resveratrol (Fig. 2b) or other aromatics (Supplementary Fig. S2), where only a minor decrease in the extracellular concentrations compared to control medium was seen.

Into the strains harboring TAL, the pathway towards resveratrol was integrated. Strains with FjTAL resulted in better titers than those expressing HaTAL (Fig. 2a, Supplementary Fig. S1a). Among the strains harboring FjTAL, ST8953 reached the highest resveratrol titer (52.1 ± 1.2 mg/L), when *VvVST1* was expressed from the strong constitutive promoter *TEFintron* (Fig. 2a, Supplementary Fig. S1a) (Tai and Stephanopoulos, 2013; Holkenbrink et al., 2018).

Next, we constructed strains capable of producing *cis*,*cis*-muconic acid and salicylic acid. For *cis*,*cis*-muconic acid, we used biosynthetic genes that have been described to work in *S. cerevisiae* (Curran et al., 2013). The engineered strain ST8959 harbored *PaAroZ* from *Podospora anserina* encoding for DHS dehydratase, three genes from *K. pneumoniae* (*KpAroY.B*, encoding FMN prenyltransferase; *KpAroY.Ciso*, enconding PCA decarboxylase; *KpAroY.D* encoding a protein that may improve PCA decarboxylase activity), and *CaCatA* from *Candida albicans* encoding catechol 1,2-dioxygenase (Fig. 1). Strain ST8959 produced 48.0 ± 6.4 mg/L *cis*,*cis*-muconic acid and 135.2 ± 0.1 mg/L of the intermediate protocatechuic acid (Fig. 2c, Supplementary Fig. S1b), suggesting that enhanced PCA decarboxylase activity could improve production, as it does in *S. cerevisiae* (Curran et al., 2013; Skjoedt et al., 2016). The salicylic acid producing strain was constructed by the expression of isochorismate synthase (EcEntC) from *E. coli* in combination with isochorismate pyruvate-lyase (PpPchB) from *Pseudomonas protegens* Pf-5 (Fig. 1), as described in *E. coli* (Lin et al., 2013). This strain resulted in 376.4 ± 12.1 mg/L salicylic acid in the broth, while no additional peaks were detected on the HPLC chromatogram with respect to the control strain, suggesting no accumulation of isochorismate (Fig. 2d, Supplementary Fig. S1c).

Among the strains described above, we observed that the titers for *cis*,*cis*-muconic acid and salicylic acid, shikimate pathway-derived products that do not require malonyl-CoA moieties, were comparable to titers in *S. cerevisiae* or *E. coli* (Curran et al., 2013; Wang et al., 2020; Lin et al., 2013). Conversely, resveratrol titers in ST8953 were 4.5 times higher than in a *S. cerevisiae* strain, when using similar biosynthetic genes and the same cultivation conditions (Li et al., 2015). These results suggest that *Y. lipolytica* has an advantage when producing compounds that require malonyl-CoA as one of the precursors, such as resveratrol. We then sought to further engineer resveratrol production in *Y. lipolytica*.

### 3.2 Feedback resistant *ARO4* and *ARO7* improved resveratrol production

**Fig. 3:**
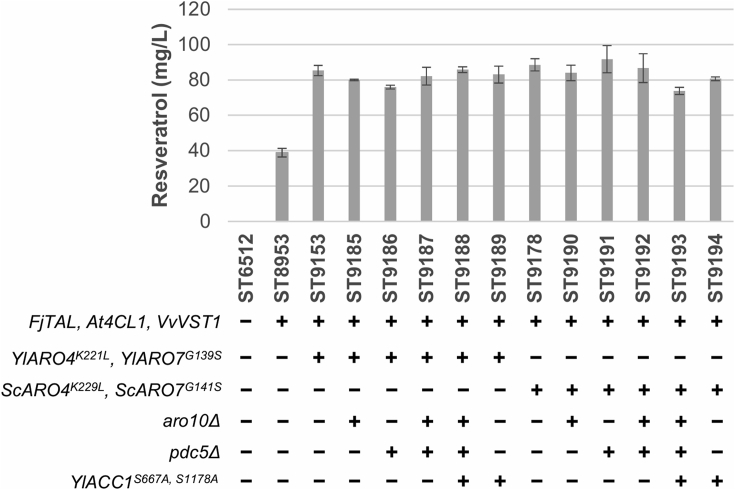
**Resveratrol titer in engineered *Y. lipolytica* strains**. Cultivations were carried out for 72 h in 24 deep-well plates containing mineral medium with 20 g/L glucose. Extracellular content was subjected to HPLC analysis. “-” and “+” symbols indicate absence or presence of the corresponding genetic modification, respectively. Error bars represent standard deviation from at least three biological replicates. *p*-Coumaric acid was not detected in any of the strains.

Several metabolic engineering targets that have been proven to work in *S. cerevisiae* were selected (Li et al., 2015). First, we introduced feedback-insensitive versions of DAHP synthase (Aro4p) and chorismate mutase (Aro7p), enzymes that are otherwise allosterically regulated by L-tyrosine (Künzler et al., 1992; Brown and Dawes, 1990). In *S. cerevisiae*, the mutant alleles *ScARO4*^*K229L*^ and *ScARO7*^*G141S*^ are common engineering targets for shikimate pathway-derived products (Luttik et al., 2008; Gold et al., 2015; Mao et al., 2017; Rodriguez et al., 2015; Li et al., 2015). More recently, it has also been demonstrated that homologous *Y. lipolytica YlARO4*^*K221L*^ allele significantly increased naringenin titer (Palmer et al., 2020). Here, we simultaneously expressed feedback insensitive alleles from *Y. lipolytica* (*YlARO4*^*K221L*^ and *YlARO7*^*G139*S^ in strain ST9153) (Supplementary Fig. S3) or *S. cerevisiae* (*ScARO4*^*K229L*^ and *ScARO7*^*G141S*^ in strain ST9178). The simultaneous introduction of mutated alleles for both genes from either of the two yeasts resulted in a similar effect, increasing resveratrol titer 2.2-fold to ca. 85 mg/L (Fig. 3, Supplementary Fig. S4). Each gene individually contributed to the increase of titer, with *ARO4* showing a larger effect (Supplementary Fig. S5).

Next, we sought to increase L-Tyr precursor supply by deleting the genes involved in the conversion of aromatic amino acids into aromatic alcohols. *ARO10* and *PDC5* deletions have been demonstrated to increase *p*-coumaric acid titers by 2-fold in *S. cerevisiae* (Rodriguez et al., 2015). We identified the *Y. lipolytica* homologous genes YALI1_D08884g (*ARO10)* and YALI1_D12832g (*PDC5*) and carried out individual and double deletions. However, none of the deletions had a positive effect on resveratrol titer (Fig. 3, Supplementary Fig. S4).

Lastly, we tried to increase malonyl-CoA supply by overexpressing *ACC1*. The last step of the pathway involves a type III PKS, which requires three malonyl-CoA units per resveratrol molecule. Thus, we hypothesized that intracellular malonyl-CoA levels could be limiting resveratrol biosynthesis. Acetyl-CoA carboxylase is the enzyme responsible for the conversion of acetyl-CoA to malonyl-CoA. In *S. cerevisiae*, acetyl-CoA carboxylase (ScAcc1p) can be phosphorylated by the kinase Snf1p in amino acids Ser659 and Ser1157, thus targeting it for degradation (Shirra et al., 2001). Two point mutations in those amino acids prevent its phosphorylation and hence results in higher malonyl-CoA supply (Shi et al., 2014). Based on homology with the *S. cerevisiae* protein, we decided to introduce the mutated *YlACC1*^*S667A, S1178A*^ (Seip et al., 2013; Kerkhoven et al., 2016). Nonetheless, expression of this mutated version of *YlACC1* did not increase resveratrol titers (Fig. 3, Supplementary Fig. S4).

### 3.3 Integration of multiple copies of resveratrol pathway led to increased resveratrol titers

**Fig. 4:**
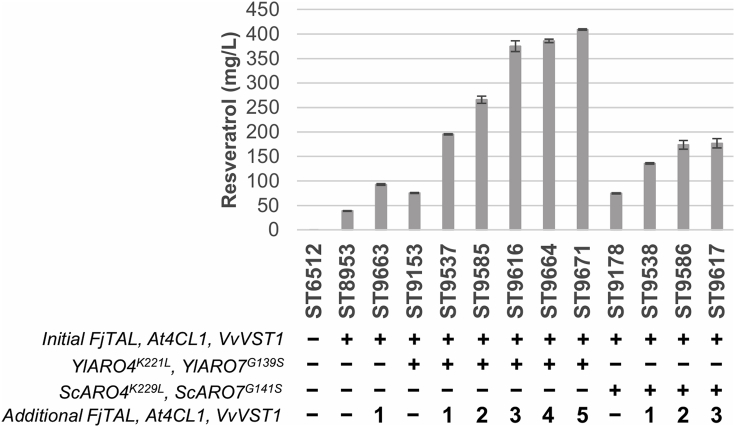
**Effect on resveratrol titers of the integration of additional copies of the heterologous pathway**. Cultivations were carried out for 72 h in 24 deep-well plates containing mineral medium with 20 g/L glucose. Extracellular content was subjected to HPLC analysis. “-” and “+” symbols indicate absence or presence of the corresponding genetic modification, respectively. Digits show the number of additional copies of resveratrol biosynthetic genes integrated. Error bars represent standard deviation from at least three biological replicates. *p*-Coumaric acid was not detected in any of the strains.

Our results suggested that precursor supply was not a significant limitation for resveratrol production at the current stage. Thus, we hypothesized that resveratrol biosynthesis could be hampered by a low activity of the heterologous pathway, with impaired capacity to convert precursors to resveratrol, which could be overcome by the integration of multiple copies of the biosynthetic genes. We first examined the effect of expressing an extra copy on a strain harboring solely the initial integration of the pathway. Strain ST9663 carrying two copies of the biosynthetic genes in the pathway resulted in a 2.4-fold increase in resveratrol titers compared to strain ST8953 (Fig. 4, Supplementary Fig. S6). We then integrated one to five additional copies of *FjTAL*, *At4CL1* and *VvVST1* into strains ST9153 and ST9178, already harboring a single copy of the pathway and feedback-resistant versions of *ARO4* and *ARO7* from *Y. lipolytica* or *S. cerevisiae*, respectively (Fig. 4, Supplementary Fig. S6, Supplementary Table S2).

In strains with *YlARO4*^*K221L*^*/YlARO7*^*G139S*^ feedback-insensitive background, the integration of one to three extra copies led to a sharp increase in resveratrol levels: 195.2 ± 1.2 (ST9537), 265.8 ± 7.4 (ST9585), and 375.1 ± 11.2 mg/L (ST9616), respectively. Addition of a fourth and fifth additional copy showed a plateauing in the titers, with only a small improvement but still reaching 409.0 ± 1.2 mg/L in ST9671, the top producing strain harboring a total of six copies. In strains expressing *S. cerevisiae* versions of the genes, improvement upon additional integrations was smaller, and saturation of the titers was observed earlier, only after two extra copies integrated. Indeed, the strain with three additional copies (ST9617, 176.8 ± 9.6 mg/L) performed worse than ST9537, with only one copy but with the mutant *ARO4*/*ARO7* sourced from *Y. lipolytica*. Taken together, these results demonstrate the higher activity of the feedback-insensitive *YlARO4*/*YlARO7* pair over its *S. cerevisiae* counterpart under this push-and-pull strategy.

### 3.4 Resveratrol production was affected by carbon source and nutrient content

**Fig. 5:**
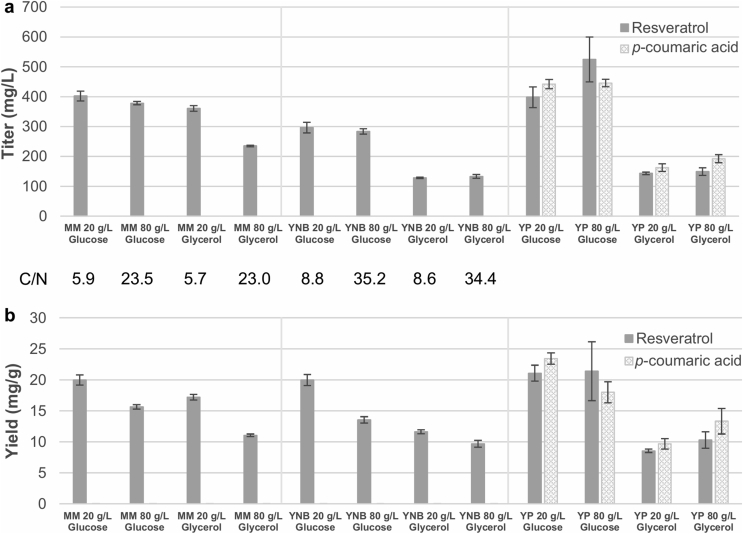
**Resveratrol production in different media.** Cultivations of strain ST9671 were carried out for 96 h in 24 deep-well plates using the media indicated for each condition. Extracellular content was subjected to HPLC analysis. Error bars represent standard deviation from at least three biological replicates. a) Titer after 96 h. b) Yield on carbon source after 96 h. MM: mineral medium, YNB: Yeast Nitrogen Base without amino acids. YP: Yeast extract Peptone. C/N stands for the molar carbon/nitrogen ratio in defined media.

In *Y. lipolytica*, medium and particularly C/N ratio have been proven to be important factors for the production of several metabolites (Jacobsen et al., 2020; Ledesma-Amaro et al., 2016). Nitrogen limitation can halt the TCA and trigger lipogenesis through ATP citrate lyase, significantly altering the flux distribution in the cells (Ratledge and Wynn, 2002). Thus, we sought to increase the resveratrol titers of our best strain ST9671 by testing different media with different C/N ratios. We evaluated defined media (mineral medium and YNB), and the rich medium YP, each of them containing either glucose or glycerol at 20 or 80 g/L and evaluated titers and yields after 96 h (Fig. 5, Supplementary Fig. S7). In addition, we assessed the cultivation profiles by sampling every 24 h (Supplementary Figs. S8–S10).

Overall, we observed that when carbon sources were supplemented at 80 g/L, only ca. 15–25 g/L were consumed after 96 h cultivation and that glucose performed better than glycerol for all types of media and concentrations (Fig. 5, Supplementary Figs. S8–S10). Cultivation in mineral medium resulted in 33–181% higher titers than in YNB medium, when comparing the experiments with the same carbon sources. The mineral medium contained 14.4 g/L of potassium phosphate monobasic, which serves as a pH buffer during the cultivation. In contrast, YNB medium contained only 1 g/L of this buffering agent and resulted in a bigger and a faster pH drop, which could have negatively influenced the growth, carbon source utilization, and resveratrol production (Supplementary Figs. S8–S9). The highest production levels were reached with rich YP with 80 g/L glucose (524.9 ± 75.1 mg/L), followed by mineral medium with 20 g/L glucose (402.2 ± 16.7 mg/L), standard medium we used for strain assessment above (Fig. 5a). However, in terms of yield on glucose, all media with 20 g/L of this substrate and YP with 80 g/L presented similar results, ca. 20 mg resveratrol/g glucose (Fig. 5b).

Interestingly, the intermediate *p*-coumaric acid was detected in significant amounts in all YP-based media, even at higher concentrations than resveratrol in most of the conditions. This was unexpected, as *p*-coumaric acid was never detected in our engineered strains in mineral medium, and was proven to be degraded in this medium by the parental strain ST6512 (Fig. 2a).

### 3.5 Fed-batch fermentation enabled high-level resveratrol production

In order to demonstrate that *Y. lipolytica* could be an industrial production host for resveratrol, we carried out a fed-batch fermentation in 1 L bioreactors with the best producing strain (ST9671). Although with YP medium resveratrol titers seemed to be better in small-scale cultivations (Fig. 5), we were concerned about *p*-coumaric acid accumulation over time (Supplementary Fig. S10), which could complicate the purification in an industrial production process. Moreover, complex medium is more expensive than mineral medium and has a higher tendency to foam. Therefore, we performed the bioreactor experiment with mineral medium. With the aim of assessing whether quantification of intracellular resveratrol in fermentation samples would be relevant, we compared the extracellular and intracellular concentration of resveratrol in a small-scale cultivation using mineral medium. Most of the resveratrol was found in the extracellular fraction (391.17 ± 4.92 mg/L), compared to 34.12 ± 3.40 mg/L intracellularly (Supplementary Fig. S11). Thus, we only measured the extracellular fraction in our fermentation samples.

**Fig. 6:**
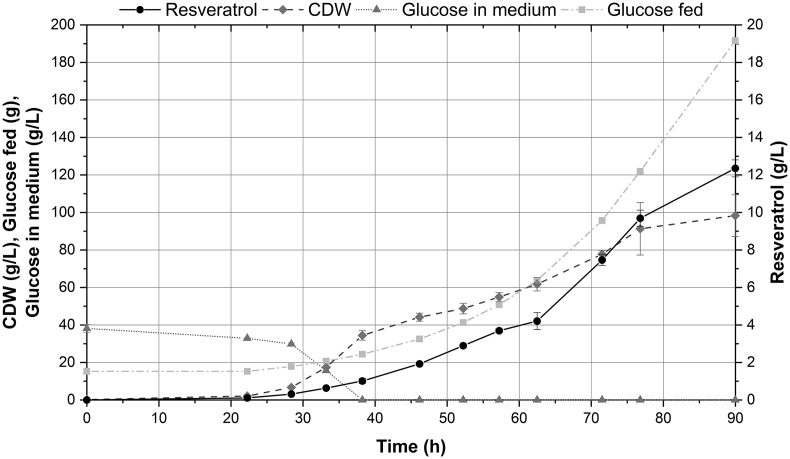
**Fed-batch fermentation in bioreactor.** Bioreactor cultivations with strain ST9671 were performed in duplicates. Data is presented as averages from both reactors, error bars represent standard deviation. Operational parameters for each of the reactors are shown in Supplementary Fig. S12. CDW: cell dry weight.

In bioreactors, cells showed a long lag phase of 22 h, probably due to the adaptation to mineral medium from YPD medium in the pre-culture. The fermentation was carried out with an exponential feeding profile, which was changed to a constant feed rate of 14 g/h for the last 14.3 h. This resulted in the addition of a total of 191.6 ± 0.8 g glucose over a span of 90 h. Controlled fermentation led to a resveratrol titer of 12.4 ± 0.3 g/L, with a yield on glucose of 54.4 ± 1.6 mg/g (15.3% of maximum theoretical yield) and a productivity of 0.14 ± 0.01 g/L/h (Fig. 6, Supplementary Fig. S12, Supplementary Fig. S13) (Vos et al., 2015). Overall, these metrics represent an improvement over the previously reported values for resveratrol production in other microbial hosts (Table 1), especially for *de novo* production. Throughout the fermentation, only small amounts of by-products like citrate and α-ketoglutarate (<80 mg/L) were detected in the batch phase, while no *p*-coumaric acid was detected at any time point (Supplementary Fig. S13). The foaming was controlled by manual addition of antifoam and in total 32.7 ± 0.1 mL of antifoam was added per L reactor volume over the course of cultivation. As this amount of antifoam is relatively high, we questioned whether the antifoaming agent could have influenced the production of resveratrol. To assess the effect of antifoam, we carried out small-scale cultivations with addition of 1, 3, and 5% v/v antifoam. The antifoam addition had a negative effect on resveratrol production at all concentrations, with decreases in titer of 41–62%. (Supplementary Fig. S14).

## 4 Discussion

In this study, high-level production of resveratrol was achieved in the emerging workhorse *Y. lipolytica*, attesting the potential of this oleaginous yeast for the production of plant secondary metabolites with a malonyl-CoA-derived structure. The strain was rationally engineered by the expression of feedback-alleviated versions of key genes in the shikimate pathway and multiple-copy integration of the genes in the heterologous pathway leading to resveratrol.

Our preliminary evaluation of the potential entailed the production of four aromatics (*p*-coumaric acid, resveratrol, salicylic acid, *cis*,*cis*-muconic acid) in the oleaginous yeast. When comparing the performance of *Y. lipolytica* with *S. cerevisiae* expressing the same pathways, a large difference was observed only for resveratrol (Fig. 2, Supplementary Fig. S1) (Curran et al., 2013; Wang et al., 2020; Lin et al., 2013). This implied that the high PPP flux in *Y. lipolytica* did not enable a high concentration of shikimate pathway intermediates, which would favor a high production of derived products like *cis*,*cis*-muconic acid or salicylic acid (Blank et al., 2005; Rodriguez et al., 2013). Therefore, a sufficient malonyl-CoA supply in the oleaginous yeast could explain the observed results with regards to resveratrol (Wasylenko et al., 2015). This initial result was in line with recent studies that used *Y. lipolytica* to produce plant-derived flavonoids like naringenin (Lv et al., 2019; Palmer et al., 2020), achieving promising initial results and the highest reported titers to date upon strain engineering. All these secondary metabolites are phenylpropanoid polyketides requiring the action of type III PKS, enzymes involved in the incorporation of malonyl-CoA to the growing molecules (Lussier et al., 2013), which could be facilitated by the native oleaginous phenotype of *Y. lipolytica*.

An important finding in our initial strain engineering steps was the degradation of *p*-coumaric acid by *Y. lipolytica* (Fig. 2b). (Palmer et al., 2020) demonstrated that concentrations up to 2 mM of *p*-coumaric acid do not affect the growth rate of *Y. lipolytica*, but they did not measure its degradation. In *S. cerevisiae*, it has been suggested that *p*-coumaric acid can be slowly converted into a range of less toxic compounds (Adeboye et al., 2015), while in other microorganisms it is transformed into *p*-hydroxybenzoic acid (Delneri et al., 1995). Since *p*-coumaric acid serves as precursor for resveratrol and many other plant phenylpropanoids, identification of the enzymes responsible for its degradation could improve the production of these metabolites. Understanding the effect of the rich medium on *p*-coumaric acid accumulation (Fig. 5) would also be informative for further strain engineering. Given that the pH in YP media never reached values as low as in mineral medium or YNB (Supplementary Figs. S8–S10), it could be hypothesized that pH could affect the export of *p*-coumaric acid out of the cells. Moreover, the presence of complex nutrients in the rich YP medium could have attenuated *p*-coumaric acid degradation. In agreement with our findings, significant production of *p*-coumaric acid has been recently achieved in *Y. lipolytica* using YP-derived medium (Gu et al., 2020a).

The rational metabolic engineering strategies that we employed to further improve resveratrol production entailed the use of feedback insensitive versions of *ARO4/ARO7*, and multiple integration of resveratrol biosynthetic genes (Fig. 3, Fig. 4). Here, we showed that *YlARO4*^*K221L*^*/YlARO7*^*G139S*^ performed better than the *S. cerevisiae* versions of these genes (Fig. 4), which could be explained by a higher activity or a lower sensitivity to feedback regulation of the enzymes from *Y. lipolytica*. In either case, this finding underlines the importance of testing several variants of key enzymes in the pathway. Our strain was further engineered by tuning the gene copy number of the heterologous pathway. Indeed, for many plant secondary metabolites, increased titers were obtained in *Y. lipolytica* by integrating multiple copies of heterologous pathway genes (Lv et al., 2019; Marella et al., 2019; Wang et al., 2016). This is most likely due to an overall low catalytic activity of the enzymes involved in secondary metabolites formation, as these compounds are typically synthesized in small amounts by the native host. With this motivation, we decided to add extra copies of the three heterologous genes, instead of examining the effect of individual genes. We also tried to increase resveratrol production by expressing a mutant version of *YlACC1* (Fig. 3), but this did not improve the titer, contrary to improvement of naringenin production in *Y. lipolytica* in a study by (Lv et al., 2019). As we later confirmed by expressing additional copies of the heterologous pathway, a plausible explanation could be that malonyl-CoA was not a limiting precursor at that engineering stage or that malonyl-CoA was used for lipids production rather than for the biosynthesis of resveratrol. Another reason could be that the phosphorylation sites are not completely conserved in *Y. lipolytica* and the enzyme became inactive upon mutations (Pomraning et al., 2016). *ARO10* and *PDC5* deletions were also performed, with no positive effect on resveratrol levels (Fig. 3), which could be caused by the low specificity of these enzymes (Vuralhan et al., 2005; Romagnoli et al., 2012) or the existence of isoenzymes.

Our top producing strain (ST9671) synthesized 409.0 ± 1.22 mg/L resveratrol *de novo* from glucose in small-scale cultivation, a 10-fold increase compared to our initial strain harboring only the biosynthetic pathway (Fig. 4). (Palmer et al., 2020) have also recently demonstrated production of resveratrol in *Y. lipolytica*. In an *ACC1*/*PEX10* overexpression background, 48.7 mg/L were produced by feeding with 2 mM *p*-coumaric acid, in a strain lacking the *TAL* gene. When *TAL* was introduced, 8.8 mg/L of resveratrol were produced *de novo* from glucose. More recently, in a work aiming at developing a *Y. lipolytica* platform strain for the production of shikimate-pathway derived products, a titer of 12.67 mg/L resveratrol was achieved (Gu et al., 2020a). In this case, the strain was initially engineered for the production of 2-phenylethanol and subsequently tested for resveratrol production. Similar to our results, the use of feedback-insensitive versions of DAHP synthases was shown to be crucial to relieve allosteric regulation of the shikimate pathway.

The fed-batch fermentation of ST9671 resulted in 12.4 ± 0.3 g/L resveratrol and a yield of 54.4 ± 1.6 mg/g, representing 15.3% of the maximum theoretical yield on glucose (354.7 mg/g) (Vos et al., 2015) (Fig. 6). This represents, to the best of our knowledge, the highest production of resveratrol and any *p*-coumaric acid-derived product in any microbial host (Thapa et al., 2019). Moreover, the fermentation was carried out in a cheap mineral medium without supplementation of any expensive aromatic intermediate or cerulenin, frequently used to inhibit lipids biosynthesis and increase malonyl-CoA pool (Lv et al., 2019; Marsafari and Xu, 2020). Further strain engineering aiming at enhancing precursors supply and a better control of foaming in the fermentation could improve the production (Fig. 4, Supplementary Fig. S14). Collectively, these results illustrate the potential of *Y. lipolytica* for a high-level production of this family of compounds.

## 5 Conclusions

In this work, we engineered the oleaginous yeast *Y. lipolytica* for the production of the plant stilbenoid resveratrol. By the integration of feedback-resistant alleles for better precursor supply and multiple copies of the biosynthetic pathway, we reached the highest resveratrol production reported to date. This contrasts with other hosts, where extensive strain engineering is needed, and illustrates the suitability of the oleaginous yeast for the production of plant secondary metabolites with a polyketide structure.

## CRediT authorship contribution statement

**Javier Sáez-Sáez:** Conceptualization, Methodology, Investigation, Validation, Writing - original draft, Writing - review & editing. **Guokun Wang:** Conceptualization, Methodology, Supervision, Writing - original draft, Writing - review & editing. **Eko Roy Marella:** Conceptualization, Methodology, Investigation, Validation. **Suresh Sudarsan:** Methodology, Investigation, Validation. **Marc Cernuda Pastor:** Investigation, Validation. **Irina Borodina:** Conceptualization, Funding acquisition, Project administration, Supervision, Writing - original draft, Writing - review & editing.

## Declaration of competing interest

The authors declare that they have no conflict of interest.
