# Supplementary material for: Engineering the oleaginous yeast Yarrowia lipolytica for high-level resveratrol production
Source: Metab Eng. 2020 Nov;62:51–61. doi: 10.1016/j.ymben.2020.08.009 (PMC7672257; doi:10.1016/j.ymben.2020.08.009)
Supplement: Multimedia component 1 [file mmc1.docx]

**Supplementary information 1**

Review of literature on microbial production of resveratrol

**Supplementary Table S1. Microbial production of resveratrol in different hosts**. Table shows different microorganisms engineered for the production of resveratrol, including metabolic engineering strategies used, titers obtained, and substrate or precursor fed. Abbreviations: *4CL*: 4-coumaroyl-CoA ligase, *STS*: stilbene synthase, *PAL*: phenylalanine ammonia-lyase, *ACC*: acetyl-CoA carboxylase, *TAL*: tyrosine ammonia-lyase; *matB*: malonyl-CoA synthetase, *matC*: malonate carrier protein, *fumC*: fumarate hydratase class II, *gapA*: glyceraldehyde-3-phosphate dehydrogenase A, *pgk*: phosphoglycerate kinase, PDH: pyruvate dehydrogenase, *fabD*: malonyl CoA-acyl carrier protein transacylase, *tktA/TKT*: transketolase 1/transketolase, *aroG*: 3-deoxy-7-phosphoheptulonate synthase, *pheA*: bifunctional chorismate mutase/prephenate dehydratase, *tyrR*: transcriptional regulatory protein, *trpE*/*D*: anthranilate synthase component 1/anthranilate phosphoribosyl transferase, *aroG/ARO3/4/5*: 3-deoxy-D-arabinoheptulosonate-7-phosphate (DAHP) synthase, *tyrA*: T-protein, *fabH*: 3-oxoacyl-[acyl-carrier-protein] synthase 3, *fabB*: 3-oxoacyl-[acyl-carrier-protein] synthase 1, *fabF*: 3-oxoacyl-[acyl-carrier-protein] synthase 2, *fabI*: enoyl-[acyl-carrier-protein] reductase [NADH] FabI, *pks*: polyketide synthase, *CPR*: cytochrome P450 reductase, *RS/VST*: resveratrol synthase, *ARO7*: chorismate mutase, *C4H*: cinnamate-4-hydroxylase, *ACS*: acetyl-CoA synthase *ATR2*: NADPH-cytochrome P450 reductase 2, *CYB5*: cytochrome b5, *ARO10*: transaminated amino acid decarboxylase, *PEX10*: peroxisomal biogenesis factor 10, *xfpK/xpkA*: phosphoketolase, *ARO1*: multifunctional AROM complex, *ARO2*: bifunctional chorismate synthase and flavin reductase, *ARO8*: aromatic aminotransferase I, *ARO9*: aromatic aminotransferase II, *TRP2*: anthranilate synthase, *TRP3*: indole-3-glycerol-phosphate synthase, *PYK*: pyruvate kinase, *PHA2*: prephenate dehydratase, fbr: feed-back resistant, *:multiple modifications, refer to publication.

| **Microbial host** | **Genetic modifications** | **Titer (mg/L)** | **Substrate/precursor** | **Reference** |
| --- | --- | --- | --- | --- |
| *E. coli* | *4CL* (*N. tabacum*)  *STS* (*V. vinifera*) | 16 | *p*-coumaric acid | (Beekwilder et al., 2006) |
| *E. coli* | *4CL* (*A. thaliana*)  *STS* (*A. hypogaea*) | 100 | *p*-coumaric acid | (Watts et al., 2006) |
| *E. coli* | *PAL* (*R. rubra*)  *4CL* (*L. erythrorhizon*)  *STS* (*A. hypogaea*)  *ACC* (*C. glutamicum*) | 37 | L-tyrosine | (Katsuyama et al., 2007a) |
| *E. coli* | *4CL* (*L. erythrorhizon*)  *STS* (*A. hypogaea*)  *ACC* (*C. glutamicum*) | 171 | *p*-coumaric acid | (Katsuyama et al., 2007b) |
| *E. coli* | *TAL* (*S. espanaensis*)  *4CL* (*S. coelicolor*)  *STS* (*A. hypogaea*) | 1.4 | *p*-coumaric acid | (Choi et al., 2011) |
| *E. coli* | *4CL* (*A. thaliana*)  *STS* (*V. vinifera*) | 2300 | *p*-coumaric acid | (Lim et al., 2011) |
| *E. coli* | *TAL* (*R. glutinis*)  *4CL* (*P. crispum*)  *STS* (*V. vinifera*)  *matB* and *matC* (*R. trifolii*) | 35.02 | L-tyrosine | (Wu et al., 2013) |
| *E. coli* | 4CL (*A. thaliana*)  STS (*V. vinifera*)  *ΔfumC*  Overexpression of *gapA*, *pgk*, PDH complex | 1600 | *p*-coumaric acid | (Bhan et al., 2013) |
| *E. coli* | *TAL* (*S. espanaensis*)  *4CL* (*S. coelicolor*)  *STS* (*A. hypogaea*) | 5.2 | Glucose | (Kang et al., 2014) |
| *E. coli* | *4CL* (*P. crispum*)  *STS* (*V. vinifera*)  *fabD* downregulation | 268.2 | *p*-coumaric acid | (Yang et al., 2015) |
| *E. coli* | *4CL* (*A. thaliana*)  *STS* (*A. hypogaea*)  (Fusion enzyme) | 80.5 | *p*-coumaric acid | (Zhang et al., 2015) |
| *E. coli* | *TAL* (*S. espanaensis*)  *4CL* (*A. thaliana*)  *STS* (*A. hypogaea*) | 114.4 | L-tyrosine | (Wang et al., 2015) |
| *E. coli* | *4CL* (*A. thaliana*)  *STS* (*A. hypogaea*) | 160 | *p*-coumaric acid | (Afonso et al., 2015) |
| *E. coli* | *TAL* (R. glutinis)  *4CL* (S. coelicolor)  *STS* (V. vinifera)  Overexpression of *tktA*^fbr^ and *aroG*^fbr^  *ΔpheA* | 22.6 | Glycerol | (Camacho-Zaragoza et al., 2016) |
| *E. coli* | *TAL* (*R. glutinis*)  *4CL* (*P. crispum*)  *STS* (*V. vinifera*)  *ΔtyrR* and *ΔtrpED* | 4.612 | Glucose | (Liu et al., 2016) |
| *E. coli* | *TAL* (*T. cutaneum*)  *4CL* (*P. crispum*)  *STS* (*V. vinifera*)  *matB* and *matC* (*R. trifolii*)  *tyrA*^fbr^ and *aroG*^fbr^ (E. coli)  Down-regulation of  *fabD*, *fabH*, *fabB*, *fabF*, *fabI*  Optimized expression of *TAL* mRNA secondary structure | 304.5 | Glucose | (Wu et al., 2017) |
| *C. glutamicum* | *STS* (*A. hypogaea*)  *4CL* (*P. crispum*)  * | 158 | *p*-coumaric acid | (Kallscheuer et al., 2016) |
| *C. glutamicum* | *TAL* (*F. johnsoniae*)  *4CL* (*P. crispum*)  *STS* (*A. hypogaea*)  *aroH* (*E. coli*)  * | 12 | Glucose | (Braga et al., 2018) |
| *L. lactis* | *TAL, 4CL, STS, ACC*  (different sources) | 1.27 | Glucose | (Gaspar et al., 2016) |
| *S. venezuelae* | *STS* (*A. hypogaea*)  *4CL* (*S. coelicolor*)  Pikromycin *pks* deletion | 0.4 | *p*-coumaric acid | (Park et al., 2009) |
| *S. cerevisiae* | *4CL* (*Populus trichocarpa × Populus deltoids*)  *STS* (*Vitis vinifera*) | 1.45  (µg/L) | *p*-coumaric acid | (Becker et al., 2003) |
| *S. cerevisiae* | *4CL* (*N. tabacum*)  *STS* (*V. vinifera*) | 6 | *p*-coumaric acid | (Beekwilder et al., 2006) |
| *S. cerevisiae* | *4CL* (*A. thaliana*)  *STS* (*V. vinifera*)  (fusion enzyme) | 5.25 | *p*-coumaric acid | (Zhang et al., 2006) |
| *S. cerevisiae* | *PAL*, *CPR* (*Populus trichocarpa × P. deltoides*)  *C4H*, *4CL* (*Glycine max*)  *RS* (*Vitis vinifera*) | 0.31 | *p*-coumaric acid | (Trantas et al., 2009) |
| *S. cerevisiae* | *4CL* (*A. thaliana*)  *STS* (*V. vinifera*) | 391 | *p*-coumaric acid | (Sydor et al., 2010) |
| *S. cerevisiae* | *TAL* (*R. sphaeroides*)  *4CL::STS (A. thaliana, V. vinífera; fusion enzyme)*  *araE* (*E. coli*) | 3.1 | *p*-coumaric acid | (Wang et al., 2011) |
| *S. cerevisiae* | *4CL1* (*A. thaliana*)  *STS* (*A. hypogaea*) | 3.1 | *p*-coumaric acid | (Shin et al., 2011) |
| *S. cerevisiae* | *PAL* (*R. toruloides*)  *4CL* (*A. thaliana*)  *STS* (*A. hypogaea*)  *ACC1* overexpression | 5.8 | L-tyrosine | (Shin et al., 2012) |
| *S. cerevisiae* | *TAL* (*H. aurantiacus*)  *4CL* (*A. thaliana*)  *VST* (*V. vinifera*)  *ARO4*^fbr^, *ARO7*^fbr^, *ACC1* | 531.41 | Ethanol | (Li et al., 2015) |
| *S. cerevisiae* | *PAL* (*A. thaliana*)  *C4H* (*A. thaliana*)  *4CL* (*A. thaliana*)  *VST* (*V. vinifera*)  *ACS* (*S. enterica*)  *ATR2* (*A. thaliana*)  Overexpression of *ARO4*^fbr^, *ARO7*^fbr^, *CYB5,* and *ACC1*  *ΔARO10* | 812 | Glucose | (Li et al., 2016) |
| *Y. lipolytica* | *PAL* (*R. glutinis*)  *4CL* (*S. coelicolor*)  *STS* (*V. vinifera*) | 1.4 | L-tyrosine | (Huang et al., 2006) |
| *Y. lipolytica* | *4CL* (*N. tabacum*)  *STS* (*A. hypogaea*)  Overexpression of *PEX10* and *ACC1* | 48.7 | *p*-coumaric acid | (Palmer et al., 2020) |
| *Y. lipolytica* | *TAL* (*R. toruloides*)  *4CL* (*P. crispum*)  *VST* (*V. vinifera*)  *ARO4*^fbr^ (*S. cerevisiae*)  *aroG*^fbr^ (*E. coli*)  *xfpK* (*B. breve*)  *xpkA* (*A. capsulatum*)  Overexpression of *ARO1*, *ARO2*, *ARO3*, *ARO4*, *ARO5*, *TKT*  Deletion of *TRP2*, *TRP3*, *ARO8*, *ARO9*, *PYK*, *PHA2* | 12.67 | Glucose | (Gu et al., 2020) |
| *Y. lipolytica* | *TAL (F. johnsoniae)*  *4CL (A. thaliana)*  *VST (V. vinifera)*  Overexpression *ARO4*^fbr^, *ARO7*^fbr^ | 12355 | Glucose | This study |

**Supplementary information 2**

Strains, plasmids, biobricks, primers and DNA fragments used in this study.

Supplementary Table S2. Strains used in this study.

| **Strain name** | **Genotype** | **Parent strain** | **Repair vector** | **gRNA vector** | **Source** |
| --- | --- | --- | --- | --- | --- |
| ST6512 | *MATa ku70∆::PrTEF1-cas9-TTef12::PrGPD-dsdAMX-TLip2 (MATa ku70∆::SpCas9-EcDsdAMX4)* | W29 (Y-63746) |  |  | (Marella et al., 2019) |
| ST8950 | *MATa ku70∆::SpCas9-EcDsdAMX4 IntC2::TPex20-PpPchB-PrGPD-PrTEFintron-EcEntC-TLip2* | ST6512 | pCfB8811 | pCfB6627 | This study |
| ST8951 | *MATa ku70∆::SpCas9-EcDsdAMX4 IntC2::PrTEFintron-FjTAL-TLip2* | ST6512 | pCfB8812 | pCfB6627 | This study |
| ST8952 | *MATa ku70∆::SpCas9-EcDsdAMX4 IntC2::PrTEFintron-HaTAL-TLip2* | ST6512 | pCfB8815 | pCfB6627 | This study |
| ST8953 | *MATa ku70∆::SpCas9-EcDsdAMX4 IntC2::PrTEFintron-FjTAL-TLip2 IntC3::TPex20-At4CL1-PrGPD-PrTEFintron-VvVST1-TLip2* | ST8951 | pCfB8816 | pCfB6630 | This study |
| ST8954 | *MATa ku70∆::SpCas9-EcDsdAMX4 IntC2::PrTEFintron-FjTAL*  *IntC3::TPex20-VvVST1-PrGPD-PrTEFintron-At4CL1-TLip2* | ST8951 | pCfB8817 | pCfB6630 | This study |
| ST8955 | *MATa ku70∆::SpCas9-EcDsdAMX4 IntC2::PrTEFintron-HaTAL-TLip2 IntC3::TPex20-At4CL1-PrGPD-PrTEFintron-VvVST1-TLip2* | ST8952 | pCfB8816 | pCfB6630 | This study |
| ST8956 | *MATa ku70∆::SpCas9-EcDsdAMX4 IntC2::PrTEFintron-HaTAL IntC3::TPex20-VvVST1-PrGPD-PrTEFintron-At4CL1-TLip2* | ST8952 | pCfB8817 | pCfB6630 | This study |
| ST8957 | *MATa ku70∆::SpCas9-EcDsdAMX4 IntC2::PrTEFintron-PaAroZ-TLip2* | ST6512 | pCfB8813 | pCfB6631 | This study |
| ST8958 | *MATa ku70∆::SpCas9-EcDsdAMX4 IntC2::PrTEFintron-PaAroZ-TLip2 IntC3::TPex20-KpAroY.D-PrGPD-PrTEFintron-KpAroY.C^iso^-TLip2* | ST8957 | pCfB8842 | pCfB6631 | This study |
| ST8959 | *MATa ku70∆::SpCas9-EcDsdAMX4 IntC2::PrTEFintron-PaAroZ-TLip2 IntC3::TPex20-KpAroY.D-PrGPD-PrTEFintron-KpAroY.C^iso^-TLip2 IntD1::TPex20-CaCatA-PrGPD-PrTEFintron-KpAroY.B-TLip2* | ST8958 | pCfB8814 | pCfB6631 | This study |
| ST9153 | *MATa ku70∆::SpCas9-EcDsdAMX4 IntC2::PrTEFintron-FjTAL-TLip2 IntC3::TPex20-At4CL1-PrGPD-PrTEFintron-VvVST1-TLip2 IntE1::TPex20-YlARO7^G139S^-PrGPD-PrTEFintron-YlARO4^K221L^-TLip2* | ST8953 | pCfB8977 | pCfB6677 | This study |
| ST9178 | *MATa ku70∆::SpCas9-EcDsdAMX4*  *IntC2::PrTEFintron-FjTAL-TLip2 IntC3::TPex20-At4CL1-PrGPD-PrTEFintron-VvVST1-TLip2 IntE1::TPex20-ScARO7^G141S^-PrGPD-PrTEFintron-ScARO4^K229L^-TLip2* | ST8953 | pCfB9009 | pCfB8858 | This study |
| ST9185 | *MATa ku70∆::SpCas9-EcDsdAMX4 IntC2::PrTEFintron-FjTAL-TLip2 IntC3::TPex20-At4CL1-PrGPD-PrTEFintron-VvVST1-TLip2 IntE1::TPex20-YlARO7^G139S^-PrGPD-PrTEFintron-YlARO4^K221L^-TLip2 aro10∆* | ST9153 | BB4039 | pCfB9010 | This study |
| ST9186 | *MATa ku70∆::SpCas9-EcDsdAMX4 IntC2::PrTEFintron-FjTAL-TLip2 IntC3::TPex20-At4CL1-PrGPD-PrTEFintron-VvVST1-TLip2 IntE1::TPex20-YlARO7^G139S^-PrGPD-PrTEFintron-YlARO4^K221L^-TLip2 pdc5∆* | ST9153 | BB4042 | pCfB9011 | This study |
| ST9187 | *MATa ku70∆::SpCas9-EcDsdAMX4 IntC2::PrTEFintron-FjTAL-TLip2 IntC3::TPex20-At4CL1-PrGPD-PrTEFintron-VvVST1-TLip2 IntE1::TPex20-YlARO7^G139S^-PrGPD-PrTEFintron-YlARO4^K221L^-TLip2 aro10∆ pdc5∆* | ST9186 | BB4039 | pCfB9015 | This study |
| ST9188 | *MATa ku70∆::SpCas9-EcDsdAMX4 IntC2::PrTEFintron-FjTAL-TLip2 IntC3::TPex20-At4CL1-PrGPD-PrTEFintron-VvVST1-TLip2 IntE1::TPex20-YlARO7^G139S^-PrGPD-PrTEFintron-YlARO4^K221L^-TLip2 aro10∆ pdc5∆ IntD1::PrTEFintron-YlACC1^S667A,S1178A^-TLip2* | ST9187 | pCfB9035 | pCfB6631 | This study |
| ST9189 | *MATa ku70∆::SpCas9-EcDsdAMX4 IntC2::PrTEFintron-FjTAL-TLip2 IntC3::TPex20-At4CL1-PrGPD-PrTEFintron-VvVST1-TLip2 IntE1::TPex20-YlARO7^G139S^-PrGPD-PrTEFintron-YlARO4^K221L^-TLip2 IntD1::PrTEFintron-YlACC1^S667A,S1178A^-TLip2* | ST9153 | pCfB9035 | pCfB6631 | This study |
| ST9190 | *MATa ku70∆::SpCas9-EcDsdAMX4*  *IntC2::PrTEFintron-FjTAL-TLip2 IntC3::TPex20-At4CL1-PrGPD-PrTEFintron-VvVST1-TLip2 IntE1::TPex20-ScARO7^G141S^-PrGPD-PrTEFintron-ScARO4^K229L^-TLip2*  *aro10∆* | ST9178 | BB4039 | pCfB9010 | This study |
| ST9191 | *MATa ku70∆::SpCas9-EcDsdAMX4*  *IntC2::PrTEFintron-FjTAL-TLip2 IntC3::TPex20-At4CL1-PrGPD-PrTEFintron-VvVST1-TLip2 IntE1::TPex20-ScARO7^G141S^-PrGPD-PrTEFintron-ScARO4^K229L^-TLip2*  *pdc5∆* | ST9178 | BB4042 | pCfB9011 | This study |
| ST9192 | *MATa ku70∆::SpCas9-EcDsdAMX4*  *IntC2::PrTEFintron-FjTAL-TLip2 IntC3::TPex20-At4CL1-PrGPD-PrTEFintron-VvVST1-TLip2 IntE1::TPex20-ScARO7^G141S^-PrGPD-PrTEFintron-ScARO4^K229L^-TLip2*  *aro10∆ pdc5∆* | ST9190 | BB4042 | pCfB9016 | This study |
| ST9193 | *MATa ku70∆::SpCas9-EcDsdAMX4*  *IntC2::PrTEFintron-FjTAL-TLip2 IntC3::TPex20-At4CL1-PrGPD-PrTEFintron-VvVST1-TLip2 IntE1::TPex20-ScARO7^G141S^-PrGPD-PrTEFintron-ScARO4^K229L^-TLip2*  *aro10∆ pdc5∆*  *IntD1::PrTEFintron-YlACC1^S667A,S1178A^-TLip2* | ST9192 | pCfB9035 | pCfB6631 | This study |
| ST9194 | *MATa ku70∆::SpCas9-EcDsdAMX4*  *IntC2::PrTEFintron-FjTAL-TLip2 IntC3::TPex20-At4CL1-PrGPD-PrTEFintron-VvVST1-TLip2 IntE1::TPex20-ScARO7^G141S^-PrGPD-PrTEFintron-ScARO4^K229L^-TLip2*  *IntD1::PrTEFintron-YlACC1^S667A,S1178A^-TLip2* | ST9178 | pCfB9035 | pCfB6631 | This study |
| ST9537 | *MATa ku70∆::SpCas9-EcDsdAMX4 IntC2::PrTEFintron-FjTAL-TLip2 IntC3::TPex20-At4CL1-PrGPD-PrTEFintron-VvVST1-TLip2 IntE1::TPex20-YlARO7^G139S^-PrGPD-PrTEFintron-YlARO4^K221L^-TLip2*  *IntE4::TPex20-FjTAL-PrTEFintron-TPex20-At4CL1-PrGPD- PrTEFintron-VvVST1-TLip2* | ST9153 | pCfB9178 | pCfB6638 | This study |
| ST9538 | *MATa ku70∆::SpCas9-EcDsdAMX4*  *IntC2::PrTEFintron-FjTAL-TLip2 IntC3::TPex20-At4CL1-PrGPD-PrTEFintron-VvVST1-TLip2 IntE1::TPex20-ScARO7^G141S^-PrGPD-PrTEFintron-ScARO4^K229L^-TLip2*  *IntE4::TPex20-FjTAL-PrTEFintron-TPex20-At4CL1-PrGPD- PrTEFintron-VvVST1-TLip2* | ST9178 | pCfB9178 | pCfB6638 | This study |
| ST9585 | *MATa ku70∆::SpCas9-EcDsdAMX4 IntC2::PrTEFintron-FjTAL-TLip2 IntC3::TPex20-At4CL1-PrGPD-PrTEFintron-VvVST1-TLip2 IntE1::TPex20-YlARO7^G139S^-PrGPD-PrTEFintron-YlARO4^K221L^-TLip2*  *IntE4,D1::TPex20-FjTAL-PrTEFintron-TPex20-At4CL1-PrGPD- PrTEFintron-VvVST1-TLip2* | ST9537 | pCfB9180 | pCfB6631 | This study |
| ST9586 | *MATa ku70∆::SpCas9-EcDsdAMX4*  *IntC2::PrTEFintron-FjTAL-TLip2 IntC3::TPex20-At4CL1-PrGPD-PrTEFintron-VvVST1-TLip2 IntE1::TPex20-ScARO7^G141S^-PrGPD-PrTEFintron-ScARO4^K229L^-TLip2*  *IntE4,D1::TPex20-FjTAL-PrTEFintron-TPex20-At4CL1-PrGPD- PrTEFintron-VvVST1-TLip2* | ST9538 | pCfB9180 | pCfB6631 | This study |
| ST9616 | *MATa ku70∆::SpCas9-EcDsdAMX4 IntC2::PrTEFintron-FjTAL-TLip2 IntC3::TPex20-At4CL1-PrGPD-PrTEFintron-VvVST1-TLip2 IntE1::TPex20-YlARO7^G139S^-PrGPD-PrTEFintron-YlARO4^K221L^-TLip2*  *IntE4,D1,E3::TPex20-FjTAL-PrTEFintron-TPex20-At4CL1-PrGPD- PrTEFintron-VvVST1-TLip2* | ST9585 | pCfB9179 | pCfB6637 | This study |
| ST9617 | *MATa ku70∆::SpCas9-EcDsdAMX4*  *IntC2::PrTEFintron-FjTAL-TLip2 IntC3::TPex20-At4CL1-PrGPD-PrTEFintron-VvVST1-TLip2 IntE1::TPex20-ScARO7^G141S^-PrGPD-PrTEFintron-ScARO4^K229L^-TLip2*  *IntE4,D1::TPex20-FjTAL-PrTEFintron-TPex20-At4CL1-PrGPD- PrTEFintron-VvVST1-TLip2* | ST9586 | pCfB9179 | pCfB6637 | This study |
| ST9663 | *MATa ku70∆::SpCas9-EcDsdAMX4 IntC2::PrTEFintron-FjTAL-TLip2 IntC3::TPex20-At4CL1-PrGPD-PrTEFintron-VvVST1-TLip2*  *IntE4::TPex20-FjTAL-PrTEFintron-TPex20-At4CL1-PrGPD- PrTEFintron-VvVST1-TLip2* | ST8953 | pCfB9178 | pCfB6638 | This study |
| ST9664 | *MATa ku70∆::SpCas9-EcDsdAMX4 IntC2::PrTEFintron-FjTAL-TLip2 IntC3::TPex20-At4CL1-PrGPD-PrTEFintron-VvVST1-TLip2 IntE1::TPex20-YlARO7^G139S^-PrGPD-PrTEFintron-YlARO4^K221L^-TLip2*  *IntE4,D1,E3,F3::TPex20-FjTAL-PrTEFintron-TPex20-At4CL1-PrGPD- PrTEFintron-VvVST1-TLip2* | ST9616 | pCfB9316 | pBP8003 | This study |
| ST9671 | *MATa ku70∆::SpCas9-EcDsdAMX4 IntC2::PrTEFintron-FjTAL-TLip2 IntC3::TPex20-At4CL1-PrGPD-PrTEFintron-VvVST1-TLip2 IntE1::TPex20-YlARO7^G139S^-PrGPD-PrTEFintron-YlARO4^K221L^-TLip2*  *IntE4,D1,E3,F3,A1::TPex20-FjTAL-PrTEFintron-TPex20-At4CL1-PrGPD-PrTEFintron-VvVST1-TLip2* | ST9664 | pCfB9315 | pBP7995 | This study |
| ST10246 | *MATa ku70∆::SpCas9-EcDsdAMX4*  *IntC2::PrTEFintron-FjTAL-TLip2 IntC3::TPex20-At4CL1-PrGPD-PrTEFintron-VvVST1-TLip2* | ST8953 | pCfB8976 | pCfB6633 | This study |
| ST10247 | *MATa ku70∆::SpCas9-EcDsdAMX4*  *IntC2::PrTEFintron-FjTAL-TLip2 IntC3::TPex20-At4CL1-PrGPD-PrTEFintron-VvVST1-TLip2* | ST8953 | pCfB9008 | pCfB6633 | This study |

Supplementary Table S3: Plasmids used in this study.

| **Plasmid** | **Description** | **Parental vector** | **BioBricks** | **Source** |
| --- | --- | --- | --- | --- |
| pCfB826 | X-4-*LoxP*-*HphMXsyn*-*ScARO7pm*<-*pTEF1-pPGK1*->*ScARO4pm* | See ref. | See ref. | (Rodriguez et al., 2015) |
| pCfB3405 | *pORI1001-Nat-CEN1-USER* | See ref. | See ref. | (Holkenbrink et al., 2018) |
| pCfB6371 | *pIntC_3-TPex20-TLip2* | See ref. | See ref. | (Holkenbrink et al., 2018) |
| pCfB6627 | *pNat-YLgRNA2_IntC_2* | See ref. | See ref. | (Holkenbrink et al., 2018) |
| pCfB6630 | *pNat-YLgRNA3_IntC_3* | See ref. | See ref. | (Holkenbrink et al., 2018) |
| pCfB6631 | *pNat-YLgRNA2_IntD_1* | See ref. | See ref. | (Holkenbrink et al., 2018) |
| pCfB6633 | *pNat-YLgRNA2_IntE_1* | See ref. | See ref. | (Holkenbrink et al., 2018) |
| pCfB6637 | *pNat-YLgRNA2_IntE_3* | See ref. | See ref. | (Holkenbrink et al., 2018) |
| pCfB6638 | *pNat-YLgRNA2_IntE_4* | See ref. | See ref. | (Holkenbrink et al., 2018) |
| pCfB6677 | *pIntE_1-TPex20-TLip2* | See ref. | See ref. | (Holkenbrink et al., 2018) |
| pCfB6682 | *pIntC_2-TPex20-TLip2* | See ref. | See ref. | (Holkenbrink et al., 2018) |
| pCfB6684 | *pIntD_1-TPex20-TLip2* | See ref. | See ref. | (Holkenbrink et al., 2018) |
| pCfB6679 | *pIntE_4-TPex20-TLip2* | See ref. | See ref. | (Holkenbrink et al., 2018) |
| pCfB6681 | *pIntE_3-TPex20-TLip2* | See ref. | See ref. | (Holkenbrink et al., 2018) |
| pCfB8681 | *pIntE_3-TPex20-PrTEFin->YlACC1->TLip2* | N/A | N/A | In-house (unpublished) |
| pCfB8811 | *pIntC_2-TPex20<-PpPchB<-PrGPD::PrTEFin->EcEntC->TLip2* | pCfB6682 | BB3855, BB3907, BB3909. BB3910 | This study |
| pCfB8812 | *pIntC_2-TPex20-PrTEFin->FjTAL->TLip2* | pCfB6682 | BB3855, BB3908, BB3911 | This study |
| pCfB8813 | *pIntC_2-TPex20-PrTEFin->PaAroZ->TLip2* | pCfB6682 | BB3855, BB3908, BB3917 | This study |
| pCfB8814 | *pIntD_1-TPex20<-CaCatA<-PrGPD::PrTEFin->KpAroY.B->TLip2* | pCfB6684 | BB3857, BB3907, BB3918, BB3921 | This study |
| pCfB8815 | *pIntC_2-TPex20-PrTEFin->HaTAL->TLip2* | pCfB6682 | BB3855, BB3908, BB3912 | This study |
| pCfB8816 | *pIntC_3-TPex20<-At4CL1<-PrGPD::PrTEFin->VvVST1->TLip2* | pCfB6371 | BB3856, BB3907, BB3913, BB3914 | This study |
| pCfB8817 | *pIntC_3-TPex20<-VvVST1<-PrGPD::PrTEFin->At4CL1->TLip2* | pCfB6371 | BB3856, BB3907, BB3915, BB3916 | This study |
| pCfB8842 | *pIntC_3-TPex20<-KpAroY.D<-PrGPD::PrTEFin->KpAroY.Ciso->TLip2* | pCfB6371 | BB3856, BB3907, BB3919, BB3920 | This study |
| pCfB8843 | *pORI1001-Hyg-CEN1-USER* | N/A | N/A | In-house  (unpublished) |
| pCfB8858 | *pHphM-YLgRNA2_IntE_1* | N/A | N/A | In-house  (unpublished) |
| pCfB8976 | *pInt E_1-TPex20-PrTEFin->YlARO4_K221L->tLip2* | pCfB6677 | BB4028, BB3908, BB4031 | This study |
| pCfB8977 | *pIntE_1-TPex20<-YlARO7G139S<-PrGPD::PrTEFin->YlARO4K221L->TLip2* | pCfB6677 | BB4028, BB3907, BB4031, BB4034 | This study |
| pCfB9008 | *pIntE_1-TPex20-PrTEFin->ScARO4_K229L->TLip2* | pCfB6677 | BB4028, BB3908, BB4035 | This study |
| pCfB9009 | *pIntE_1-TPex20<-ScARO7G141S<-PrGPD::PrTEFin->ScARO4K229L->TLip2* | pCfB6677 | BB4028, BB3907, BB4035, BB4036 | This study |
| pCfB9010 | *pNat-gRNA-YALI1_D08884g* | pCfB3405 | BB4050 | This study |
| pCfB9011 | *pNat-gRNA-YALI1_D12832g* | pCfB3405 | BB4051 | This study |
| pCfB9015 | *pHphM-gRNA-YALI1_D08884g* | pCfB8843 | BB4050 | This study |
| pCfB9016 | *pHphM-gRNA-YALI1_D12832g* | pCfB8843 | BB4051 | This study |
| pCfB9035 | *pIntD_1-TPex20-PrTEFin->YlACC1_S667A+S1178A->TLip2* | pCfB6684 | BB3857, BB4045, BB4049 | This study |
| pCfB9178 | *pIntE_4-TPex20<-FjTAL<-PrTEFin,*  *<-TPex20<-At4CL1-PrGPD,PrTEFin->VvVST1->TLip2->* | pCfB6679 | BB4154, BB4156, BB4157 | This study |
| pCfB9179 | *pIntE_3-TPex20<-FjTAL<-PrTEFin*  *<-TPex20<-At4CL1-PrGPD,PrTEFin->VvVST1->TLip2->* | pCfB6681 | BB4153, BB4156, BB4157 | This study |
| pCfB9180 | *pIntD_1-TPex20<-FjTAL<-PrTEFin,*  *<-TPex20<-At4CL1-PrGPD,PrTEFin->VvVST1->TLip2->* | pCfB6684 | BB3857, BB4156, BB4157 | This study |
| pCfB9315 | *pIntA_1-TPex20<-FjTAL<-PrTEFin,*  *<-TPex20<-At4CL1-PrGPD,PrTEFin->VvVST1->TLip2->* | pBP8006 | BB4322, BB4156, BB4157 | This study |
| pCfB9316 | *pIntF_3-TPex20<-FjTAL<-PrTEFin,*  *<-TPex20<-At4CL1-PrGPD,PrTEFin->VvVST1->TLip2->* | pBP8009 | BB4323, BB4156, BB4157 | This study |
| pBP7995 | *pNat-YLgRNA5-IntA_1* | N/A | N/A | BioPhero ApS |
| pBP8003 | *pNat-YLgRNA4-IntF_3* | N/A | N/A | BioPhero ApS |
| pBP8006 | *pIntA_1-TPex20-TLip2* | N/A | N/A | BioPhero ApS |
| pBP8009 | *pIntF_3-TPex20-TLip2* | N/A | N/A | BioPhero ApS |

Supplementary Table S4: Biobricks used in this study.

| **BioBrick** | **Description** | **PCR template** | **Forward primer** | **Reverse primer** |
| --- | --- | --- | --- | --- |
| BB3855 | pIntC_2 backbone | pCfB6682 | PR-23963 | PR-23968 |
| BB3856 | pIntC_3 backbone | pCfB6371 | PR-23963 | PR-23968 |
| BB3857 | pIntD_1 backbone | pCfB6684 | PR-23963 | PR-23968 |
| BB3905 | *PrTEFin*-> | gDNA ST6512 | PR-23966 | PR-23967 |
| BB3906 | <-*PrGPD* | gDNA ST6512 | PR-23964 | PR-23965 |
| BB3907 | <-*PrGPD::PrTEFin*-> | BB3905+BB3906 | PR-23965 | PR-23967 |
| BB3908 | *PrTEFin*-> | gDNA ST6512 | PR-23995 | PR-23967 |
| BB3909 | <-*PpPchB* | *PpPchB* GeneArt DNA String | PR-23973 | PR-23974 |
| BB3910 | *EcEntC*-> (*PrTEFin*) | *EcEntC* GeneArt DNA String | PR-24286 | PR-23976 |
| BB3911 | *FjTAL*-> (*PrTEFin*) | *FjTAL* GeneArt DNA String | PR-24287 | PR-23997 |
| BB3912 | *HaTAL*-> (*PrTEFin*) | *HaTAL* GeneArt DNA String | PR-24290 | PR-23999 |
| BB3913 | <-*At4CL1* | *At4CL1* GeneArt DNA String | PR-23977 | PR-23978 |
| BB3914 | *VvVST1*-> (*PrTEFin*) | *VvVST1* GeneArt DNA String | PR-24291 | PR-23980 |
| BB3915 | <-*VVVST1* | *VvVST1* GeneArt DNA String | PR-23983 | PR-23984 |
| BB3916 | *At4CL1*-> (*PrTEFin*) | *At4CL1* GeneArt DNA String | PR-24292 | PR-23982 |
| BB3917 | *PaAroZ*-> (*PrTEFin*) | *PaAroZ* GeneArt DNA String | PR-24288 | PR-24002 |
| BB3918 | *KpAroY.B*-> (*PrTEFin*) | *KpAroY.B* GeneArt DNA String | PR-24289 | PR-23994 |
| BB3919 | *KpAroY.Ciso*-> (*PrTEFin*) | *KpAroY.Ciso* GeneArt DNA String | PR-24293 | PR-23989 |
| BB3920 | <-*KpAroY.D* | *KpAroY*.*D* GeneArt DNA String | PR-23986 | PR-23987 |
| BB3921 | <-*CaCatA* | *CaCatA* GeneArt DNA String | PR-23991 | PR-23992 |
| BB4028 | pIntE_1 backbone | pCfB6677 | PR-23963 | PR-23968 |
| BB4029 | *YlARO4K229L*, part 1 | gDNA ST6512 | PR-24549 | PR-24550 |
| BB4030 | *YlARO4K229L*, part 2 | gDNA ST6512 | PR-24551 | PR-24552 |
| BB4031 | *YlARO4K229L*-> (*PrTEFin*) | BB4029+BB4030 | PR-24549 | PR-24552 |
| BB4032 | *YlARO7G139S*, part 1 | gDNA ST6512 | PR-24553 | PR-24554 |
| BB4033 | *YlARO7G139S*, part 2 | gDNA ST6512 | PR-24555 | PR-24556 |
| BB4034 | <-*YlARO7G139S* | BB4032+BB4033 | PR-24553 | PR-24556 |
| BB4035 | *ScARO4K229L*-> (*PrTEFin*) | pCfB826 | PR-24589 | PR-24590 |
| BB4036 | <-*ScARO7G141S* | pCfB826 | PR-24591 | PR-24592 |
| BB4037 | *ARO10*_Up | gDNA ST6512 | PR-24567 | PR-24568 |
| BB4038 | *ARO10*_Down | gDNA ST6512 | PR-24569 | PR-24570 |
| BB4039 | *ARO10*_FullRepair | BB4037+BB4038 | PR-24567 | PR-24570 |
| BB4040 | *PDC5*_Up | gDNA ST6512 | PR-24575 | PR-24576 |
| BB4041 | *PDC5*_Down | gDNA ST6512 | PR-24577 | PR-24578 |
| BB4042 | *PDC5*_FullRepair | BB4040+BB4041 | PR-24575 | PR-24578 |
| BB4043 | PrTEFin+*YlACC1*-Ex1 | pCfB8681 | PR-23995 | PR-24667 |
| BB4044 | *YlACC1*-Ex2 | gDNA ST6512 | PR-24668 | PR-24669 |
| BB4045 | *PrTEFin*+*YlACC1*-Ex1-Ex2 | BB4043+4044 | PR-23995 | PR-24669 |
| BB4046 | *YlACC1*-Ex3-Part1 | gDNA ST6512 | PR-24670 | PR-18559 |
| BB4047 | *YlACC1*-Ex3-Part2 | gDNA ST6512 | PR-18558 | PR-18561 |
| BB4048 | *YlACC1*-Ex3-Part3 | gDNA ST6512 | PR-18560 | PR-24671 |
| BB4049 | *YlACC1*-Ex3-S667A,S1178A | BB4046+BB4047+BB4048 | PR-24670 | PR-24671 |
| BB4050 | gRNA-*ARO10*-single | BB1635+BB1636+PR-24565+PR-24566 | PR-10607 | PR-10604 |
| BB4051 | gRNA-*PDC5*-single | BB1635+BB1636+PR-24567+PR-24568 | PR-10607 | PR-10604 |
| BB4153 | pIntE_3 backbone | pCfB6681 | PR-23963 | PR-23968 |
| BB4154 | pIntE_4 backbone | pCfB6679 | PR-23963 | PR-23968 |
| BB4155 | pIntF_3 backbone | pBP8009 | PR-23963 | PR-23968 |
| BB4156 | <-*FjTAL*-*PrTEFin* | pCfB8812 | PR-25054 | PR-25055 |
| BB4157 | <*At4CL1*-*PrGPD*::*PrTEFin*->*VvVST1*->) | pCfB8816 | PR-25056 | PR-23980 |
| BB4322 | pIntA_1 backbone | pBP8006 | PR-23963 | PR-23968 |

Table S5: Primers used in this study.

| **Primer** | **Sequence** | **Description** |
| --- | --- | --- |
| PR-10604 | cacgcgaUaccgtacccacacaaaaaaagcaccaccgactc | Reverse primer for the amplification of crRNA-TRPR |
| PR-10607 | cgtgcgaUagtgaatcattgctaacagatc | Forward primer for the amplification of PrtRNAGly |
| PR-15788 | taaccaaccUgcgccgacccggaatcgaac | Reverse primer for the amplification of PrtRNAGly |
| PR-15789 | gttttagagcUagaaatagcaagttaaaataag | Forward primer for the amplification of crRNA-TRPR |
| PR-15790 | AGTGCAGGUagtgaatcattgctaacagatc | Forward primer for the amplification of gRNA cassette in position 2 |
| PR-15791 | ACCTGCACUaccgtacccacacaaaaaaagcac | Reverse primer for the amplification of gRNA cassette in position 2 |
| PR-18558 | ACCTCTTGCuGACGGTGGTATTCTGTGTCT | Forward primer for YlACC1 point mutation 1 in exon 3 |
| PR-18559 | AGCAAGAGGuCTAACTCCAATGTCGCATCG | Reverse primer for YlACC1 point mutation 1 in exon 3 |
| PR-18560 | ATGCCGTCuCCGACTTTTCGTACACCGTT | Forward primer for YlACC1 point mutation 2 in exon 3 |
| PR-18561 | AGACGGCAuCAGCTCGAGACACCGAGG | Reverse primer for YlACC1 point mutation 2 in exon 3 |
| PR-23963 | ATCGCACGuAAGTGTGGATGGGGAAGTGAGT | Forward primer for amplification of EasyCloneYALI integrative vectors |
| PR-23964 | atcagtagcuGACGCAGTAGGATGTCCTGCA | Forward primer for amplification of *PrGPD*, position 1. Double promoter |
| PR-23965 | ACCTGCACuTGTTGATGTGTGTTTAATTCAAGAATGAAT | Reverse primer for amplification of *PrGPD*, position 1. Double promoter |
| PR-23966 | agctactgauAGAGACCGGGTTGGCGGC | Forward primer for amplification of *PrTEFin*, position 2. Double promoter |
| PR-23967 | AGTACTGCAAAAAGUGCTGGTCGGA | Reverse primer for amplification of *PrTEFin*, position 2. Double promoter |
| PR-23968 | atcgcgtguCTTCTGTTCGGAATCAACCTCAAGG | Reverse primer for amplification of EasyCloneYALI integrative vectors |
| PR-23973 | ACGTGCGAuTTACTCGTCCTGGGCGCCC | Reverse primer for amplification of *PpPchB* in position 1 |
| PR-23974 | AGTGCAGGuGCCACAATGAACTTCCCTCTGGTGGACC | Forward primer for amplification of *PpPchB* in position 1 |
| PR-23976 | aCACGCGAuTTAGTGCAGGCCGAACACGT | Reverse primer for amplification of *EcEntC* in position 2 |
| PR-23977 | AGTGCAGGuGCCACAATGGCTCCCCAAGAGCAGG | Forward primer for amplification of *At4CL1* in position 1 |
| PR-23978 | ACGTGCGAuTTACAGGCCGTTAGCCAGCT | Reverse primer for amplification of *At4CL1* in position 1 |
| PR-23980 | acacgcgauTTAGTTGGTCACGGTGGGCAC | Reverse primer for amplification of *VvVST1* in position 2 |
| PR-23982 | acacgcgauTTACAGGCCGTTAGCCAGCTT | Reverse primer for amplification of *At4CL1* in position 2 |
| PR-23983 | AGTGCAGGuGCCACAATGGCCTCTGTGGAAGAGTTCC | Forward primer for amplification of *VvVST1* in position 1 |
| PR-23984 | ACGTGCGAuTTAGTTGGTCACGGTGGGCA | Reverse primer for amplification of VvVST1 in position 1 |
| PR-23986 | AGTGCAGGuGCCACAATGATCTGTCCCCGATGCG | Forward primer for amplification of *KpAroY.D* in position 1 |
| PR-23987 | ACGTGCGAuTTATCGCTTGTCCTCGGGCAG | Reverse primer for amplification of *KpAroY.D* in position 1 |
| PR-23989 | acacgcgauTTACTTGGCAGAGCCCTGGT | Reverse primer for amplification of *KpAroY.Ciso* in position 2 |
| PR-23991 | AGTGCAGGuGCCACAATGTCTCAGGCCTTCACCGA | Forward primer for amplification *CaCatA* in position 1 |
| PR-23992 | ACGTGCGAuTTACAGCTTGATCTCGGCGTCC | Reverse primer for amplification of *CaCatA* in position 1 |
| PR-23994 | acacgcgauTTACTCGATCTCTTGGGCGAACTG | Reverse primer for amplification of *KpAroY.B* in position 2 |
| PR-23995 | ACGTGCGAuAGAGACCGGGTTGGCGGC | Forward primer for amplificatio of *PrTEFin* in position 2, single promoter |
| PR-23997 | acacgcgauCTAGTTGTTAATCAGATGGTCCTTGACCTTC | Reverse primer for amplification of *FjTAL* in position 2 |
| PR-23999 | acacgcgauTTATCGGAACAGGATGATGGATCGC | Reverse primer for amplification of *HaTAL* in position 2 |
| PR-24002 | acacgcgauTTACAGGGCAGCAGACAGAGAC | Reverse primer for amplification of *PaAroZ* in position 2 |
| PR-24286 | ACTTTTTGCAGTACUAACCGCAGGACACCTCTCTGGCCGAAGAG | Forward primer for amplification of *EcEntC* in position 2, under control of *PrTEFin* |
| PR-24287 | ACTTTTTGCAGTACUAACCGCAGAACACCATCAACGAGTACCTGTCT | Forward primer for amplification of *FjTAL* in position 2, under control of *PrTEFin* |
| PR-24288 | ACTTTTTGCAGTACUAACCGCAGCCCTCTAAGCTGGCCATCAC | Forward primer for amplification of *PaAroZ* in position 2, under control of *PrTEFin* |
| PR-24289 | ACTTTTTGCAGTACUAACCGCAGAAGCTGATCATCggtatgactggt | Forward primer for amplification of *KpAroY.B* in position 2, under control of *PrTEFin* |
| PR-24290 | ACTTTTTGCAGTACUAACCGCAGTCTACCACTCTGATCCTGACCGG | Forward primer for amplification of HaTAL in position 2, under control of PrTEFin |
| PR-24291 | ACTTTTTGCAGTACUAACCGCAGGCCTCTGTGGAAGAGTTCCGA | Forward primer for amplification of *VvVST1* in position 2, under control of *PrTEFin* |
| PR-24292 | ACTTTTTGCAGTACUAACCGCAGGCTCCCCAAGAGCAGGCC | Forward primer for amplification of *At4CL1* in position 2, under control of *PrTEFin* |
| PR-24293 | ACTTTTTGCAGTACUAACCGCAGACCGCTCCTATCCAGGACC | Forward primer for amplification of *KpAroY.Ciso* in position 2, under control of *PrTEFin* |
| PR-24549 | ACTTTTTGCAGTACUaaccgcagtcccgttcctcctctcccaac | Forward primer for amplification of *YlARO4* part 1 with point mutation |
| PR-24550 | acacccaUgaagtggtgagggt | Reverse primer for amplification of *YlARO4* part 1 with point mutation |
| PR-24551 | atgggtgUcaccctgcagggtgttgccgccatc | Forward primer for amplification of *YlARO4* part 2 with point mutation |
| PR-24552 | acacgcgaUttagttcttgtttcgtcgctcct | Reverse primer for amplification of *YlARO4* part 2 with point mutation |
| PR-24553 | AGTGCAGGUGCCACAATGGACTTCACTAAAGCCGACACC | Forward primer for amplification of *YlARO7* part 1 with point mutation |
| PR-24554 | ACCGAGGAAUAGTTCTCGGGCTGATCTCCA | Reverse primer for amplification of *YlARO7* part 1 with point mutation |
| PR-24555 | ATTCCTCGGUCATGGTGTGCGACATC | Forward primer for amplification of *YlARO7* part 2 with point mutation |
| PR-24556 | ACGTGCGAUCTACTCCAACCGCCGGAGCA | Reverse primer for amplification of *YlARO7* part 2 with point mutation |
| PR-24565 | GAAGTGGACTAAGTTGCTGGgttttagagct | gRNA forward primer for *ARO10* |
| PR-24566 | CCAGCAACTTAGTCCACTTCtaaccaacct | gRNA reverse primer for *ARO10* |
| PR-24567 | TGCATCTGGCTCTAAATGTCTCC | Forward primer for amplification of *ARO10* upstream homology arm |
| PR-24568 | ATGTTGAUAGTCAAGTCACTGGAGAGATG | Reverse primer for amplification of *ARO10* upstream homology arm |
| PR-24569 | ATCAACAUGATGAGATTGAGCGGAGCG | Forward primer for amplification of *ARO10* downstream homology arm |
| PR-24570 | TCGGCTACCGGTTTGACTCAC | Reverse primer for amplification of *ARO10* downstream homology arm |
| PR-24573 | TGTACAATGTCGACATGCGGgttttagagct | gRNA forward primer for *PDC5* |
| PR-24574 | CCGCATGTCGACATTGTACAtaaccaacct | gRNA reverse primer for *PDC5* |
| PR-24575 | TCAGGCGTTGCTTTTGCTCC | Forward primer for amplification of *PDC5* upstream homology arm |
| PR-24576 | ATGCTGGTUAGCAAGTCAAGTTAGGTTTTGGG | Reverse primer for amplification of *PDC5* upstream homology arm |
| PR-24577 | AACCAGCAUTATAGATGAATCATTTAAAAAGAGATGACTTGTAG | Forward primer for amplification of *PDC5* downstream homology arm |
| PR-24578 | TTCGCCATTCCCGCTCATTC | Reverse primer for amplification of *PDC5* downstream homology arm |
| PR-24589 | ACTTTTTGCAGTACUaaccgcagAGTGAATCTCCAATGTTCGCTGC | Forward primer for amplification of *ScARO4K229L* in position 2 with *PrTEFin* |
| PR-24590 | acacgcgaUTCATTTCTTGTTAACTTCTCTTCTTTGTCTGACAG | Reverse primer for amplification of *ScARO4K229L* in position 2 with *PrTEFin* |
| PR-24591 | AGTGCAGGUGCCACAatggatttcacaaaaccagaaactgttttaaatc | Forward primer for amplification of *ScARO7G141S* in position 1 |
| PR-24592 | ACGTGCGAUttactcttccaaccttcttagcaagtattcc | Reverse primer for amplification of *ScARO7G141S* in position 1 |
| PR-24667 | AGCCATACUGAAAAACCGACGTGTTAGTGTCC | Reverse primer for amplification of exon 1 in *YlACC1* |
| PR-24668 | AGTATGGCUTCAGGATCTTCAACGC | Forward primer for amplification of exon 2 in *YlACC1* |
| PR-24669 | ATGAGGACCUTGTTGATAACTGTATGACCTCCGTG | Reverse primer for amplification of exon 2 in *YlACC1* |
| PR-24670 | AGGTCCTCAUCGCTAACAACGGTATTG | Forward primer for amplification of exon 3 in *YlACC1* |
| PR-24671 | ACACGCGAUTCACAACCCCTTGAGCAGCTC | Reverse primer for amplification of exon 3 in *YlACC1* |
| PR-25054 | acgtgcgaUctagttgttaatcagatggtccttgaccttc | Forward primer to amplify fused *PrTEFin-FjTAL* |
| PR-25055 | AGCATAAGUagagaccgggttggcggc | Rerverse primer to amplify *fused PrTEFin-FjTAL* |
| PR-25056 | ACTTATGCUacgcaactaacatgaatgaatacgatatacatc | Forward primer to amplify whole expression cassette from pCfB8816 |

Supplementary Table S6: Codon-optimized genes for *Y. lipolytica* used in this study.

| *HaTAL* | ATGTCTACCACTCTGATCCTGACCGGCGAAGGCCTGGGCATCGACGACGTGGTGCGAGTGGCCCGACACCAGGACCGAGTCGAGCTGACCACCGATCCTGCCATTCTGGCCCAGATCGAGGCCTCTTGCGCCTACATCAACCAGGCCGTGAAGGAACACCAGCCTGTGTACGGCGTGACCACCGGCTTCGGCGGCATGGCCAACGTGATCATCTCTCCCGAGGAAGCCGCCGAGCTGCAGAACAACGCCATCTGGTATCACAAGACCGGCGCTGGCAAGCTGCTGCCCTTCACCGACGTGCGAGCCGCCATGCTGCTGCGAGCCAACTCTCACATGCGAGGTGCCTCTGGCATCCGACTCGAGATCATCCAGCGAATGGTGACCTTCCTGAACGCCAACGTCACCCCTCACGTGCGAGAGTTCGGCTCTATCGGCGCCTCTGGCGATCTGGTGCCCCTGATCTCTATCACCGGCGCTCTGCTGGGCACCGACCAGGCTTTCATGGTGGACTTCAACGGCGAGACTCTGGACTGCATCTCTGCCCTCGAGCGACTGGGACTGCCCCGACTGCGACTGCAGCCCAAGGAAGGACTGGCCATGATGAACGGCACCTCTGTGATGACCGGAATCGCCGCCAACTGCGTGCACGACGCCCGAATCCTGCTGGCCCTGGCTCTCGAGGCCCACGCTCTGATGATCCAGGGACTGCAGGGCACTAACCAGTCTTTTCACCCCTTCATCCACCGACACAAGCCCCACACCGGACAGGTGTGGGCCGCTGACCACATGCTCGAGCTGCTGCAGGGCTCTCAGCTGTCTCGAAACGAGCTGGACGGCTCTCACGACTACCGAGATGGCGACCTGATTCAGGACCGATACTCTCTCCGATGTCTGCCCCAGTTCCTGGGACCTATCATCGACGGAATGGCCTTCATCTCTCACCATCTGCGAGTCGAGATCAACTCTGCCAACGACAACCCTCTGATCGACACCGCCTCTGCCGCCTCTTACCACGGCGGCAACTTTCTGGGACAGTACATCGGCGTCGGCATGGACCAGCTGCGGTACTACATGGGCCTGATGGCCAAGCACCTGGACGTGCAGATCGCCCTGCTGGTGTCTCCTCAGTTCAACAACGGACTGCCCGCCTCTCTGGTGGGCAACATTCAGCGAAAGGTGAACATGGGACTGAAGGGCCTGCAGCTGACCGCCAACTCTATTATGCCTATCCTGACCTTTCTGGGCAACTCTCTGGCCGATCGATTCCCCACTCACGCCGAGCAGTTCAACCAGAACATCAACTCTCAAGGCTTCGGATCTGCCAACCTGGCTCGACAGACCATTCAGACCCTCCAGCAGTACATTGCTATCACCCTGATGTTCGGCGTGCAGGCCGTGGACCTGCGAACCCACAAGCTGGCCGGACACTACAACGCCGCTGAGCTGCTGTCTCCCCTGACCGCTAAGATCTACCACGCCGTGCGATCTATCGTGAAGCACCCTCCTAGTCCTGAGCGACCCTACATCTGGAACGACGACGAGCAGGTCCTCGAGGCTCACATCTCTGCTCTGGCCCACGATATCGCCAACGACGGATCTCTGGTGTCTGCCGTCGAGCAGACCCTGTCTGGCCTGCGATCCATCATCCTGTTCCGATAA |
| --- | --- |
| *FjTAL* | ATGAACACCATCAACGAGTACCTGTCTCTGGAAGAGTTCGAGGCCATCATCTTCGGCAACCAGAAGGTGACCATCTCTGACGTGGTGGTGAACCGAGTGAACGAGTCTTTCAACTTCCTGAAGGAATTCTCTGGCAACAAGGTGATCTACGGCGTGAACACCGGCTTCGGCCCCATGGCTCAGTACCGAATCAAGGAATCTGACCAGATCCAGCTGCAGTACAACCTGATCCGATCTCACTCTTCTGGCACCGGCAAGCCTCTGTCTCCCGTGTGCGCCAAGGCCGCCATTCTGGCCCGACTGAACACCCTGTCGCTGGGCAACTCTGGCGTGCACCCCTCTGTGATCAACCTGATGTCTGAGCTGATCAACAAGGACATTACCCCTCTGATCTTCGAGCACGGCGGCGTGGGCGCCTCTGGCGACCTGGTGCAGCTGTCTCACCTGGCTCTGGTGCTGATCGGCGAGGGCGAAGTGTTCTACAAGGGCGAGCGACGACCCACTCCTGAGGTGTTCGAGATCGAGGGACTGAAGCCCATCCAGGTCGAGATCCGAGAGGGACTCGCCCTGATCAACGGCACCTCCGTGATGACCGGCATCGGCGTGGTGAACGTGTACCACGCCAAGAAGCTGCTGGACTGGTCCCTGAAGTCCTCTTGCGCCATTAACGAGCTGGTGCAGGCCTACGACGACCACTTCTCTGCCGAGCTGAACCAGACCAAGCGACACAAGGGCCAGCAAGAGATCGCCCTGAAGATGCGACAGAACCTGTCTGACTCTACCCTGATTCGAAAGCGAGAGGACCACCTGTACTCTGGCGAGAACACCGAGGAAATCTTCAAGGAAAAGGTGCAAGAGTACTACTCTCTCCGATGCGTGCCCCAGATTCTGGGCCCCGTGCTGGAAACCATCAACAACGTGGCCTCTATTCTCGAGGACGAGTTCAACTCTGCCAACGACAACCCCATCATCGACGTGAAGAACCAGCACGTCTACCACGGCGGCAACTTCCACGGCGACTACATCTCCCTCGAGATGGACAAGCTGAAGATCGTGATCACCAAGCTGACCATGCTGGCCGAGCGACAGCTGAACTACCTGCTGAACTCTAAGATCAACGAGCTGCTGCCTCCTTTCGTGAACCTGGGCACCCTGGGCTTCAACTTCGGCATGCAGGGCGTGCAGTTCACCGCCACCTCTACCACCGCCGAGTCTCAGATGCTGTCTAACCCCATGTACGTGCACTCTATCCCCAACAACAACGATAACCAGGACATCGTGTCTATGGGCACCAACTCCGCCGTGATTACCTCTAAGGTGATCGAGAACGCCTTCGAGGTGCTGGCCATCGAGATGATCACCATCGTGCAGGCCATTGACTACCTGGGCCAGAAGGACAAGATCTCTTCTGTGTCTAAGAAGTGGTACGACGAGATTCGAAACATCATCCCCACCTTTAAGGAAGATCAGGTGATGTACCCCTTCGTGCAGAAGGTCAAGGACCATCTGATTAACAACTAG |
| *At4CL1* | ATGGCTCCCCAAGAGCAGGCCGTGTCTCAGGTGATGGAAAAGCAGTCTAACAACAACAACTCTGACGTGATCTTCCGATCTAAGCTGCCCGACATCTACATCCCCAACCACCTGTCTCTGCACGACTACATCTTCCAGAACATCTCTGAGTTCGCCACCAAGCCTTGCCTGATCAACGGCCCCACCGGCCACGTGTACACCTACTCCGACGTCCACGTGATCTCTCGACAGATCGCCGCCAACTTCCACAAGCTGGGCGTGAACCAGAACGACGTGGTGATGCTGCTGCTGCCCAACTGTCCCGAGTTCGTGCTGTCTTTCCTGGCCGCCTCGTTCCGAGGCGCCACCGCTACCGCTGCTAACCCATTCTTCACCCCTGCCGAGATCGCCAAGCAGGCCAAGGCCTCTAACACCAAGCTGATCATCACCGAGGCTCGATACGTGGACAAGATCAAGCCCCTGCAGAACGATGACGGCGTGGTGATCGTGTGCATCGACGACAACGAGTCTGTGCCCATTCCTGAGGGCTGCCTGCGATTCACCGAGCTGACCCAGTCTACCACCGAGGCCTCTGAGGTGATCGACTCCGTCGAGATCTCTCCCGACGACGTTGTGGCTCTGCCCTACTCTTCTGGCACCACCGGACTGCCCAAGGGCGTGATGCTGACCCACAAGGGCCTCGTGACCTCTGTGGCCCAGCAGGTCGACGGCGAGAACCCCAACCTGTACTTCCACTCTGACGACGTGATCCTGTGCGTGCTGCCCATGTTCCACATCTACGCCCTGAACTCTATCATGCTGTGCGGCCTGCGAGTGGGAGCCGCCATCCTGATCATGCCCAAGTTCGAGATCAACCTGCTGCTCGAGCTGATCCAGAGATGCAAGGTGACCGTGGCTCCTATGGTGCCTCCTATCGTGCTGGCCATTGCCAAGTCCTCTGAGACTGAGAAGTACGACCTGTCCTCTATCCGAGTGGTGAAGTCTGGCGCTGCTCCCCTCGGCAAGGAACTCGAGGACGCCGTGAACGCTAAGTTCCCCAACGCCAAGCTCGGACAAGGCTACGGCATGACCGAGGCTGGCCCCGTCCTGGCCATGTCTCTGGGCTTCGCCAAGGAACCCTTTCCAGTCAAGTCCGGCGCCTGCGGCACCGTGGTGCGAAACGCCGAGATGAAGATCGTGGACCCCGACACCGGCGACTCCCTGTCTCGAAACCAGCCTGGCGAGATCTGCATCCGAGGCCACCAGATCATGAAGGGCTACCTGAACAACCCCGCTGCCACCGCCGAGACTATCGACAAGGACGGCTGGCTGCACACCGGTGACATCGGCCTGATTGACGACGACGATGAGCTGTTCATTGTGGACCGACTGAAGGAACTGATCAAGTACAAGGGCTTCCAGGTGGCTCCCGCCGAGCTTGAGGCCCTGCTGATCGGACACCCCGACATCACCGACGTGGCCGTGGTCGCCATGAAGGAAGAGGCCGCTGGCGAGGTGCCCGTGGCCTTCGTGGTCAAGTCTAAGGACTCTGAGCTGTCTGAGGACGACGTCAAGCAGTTCGTGTCTAAGCAGGTCGTGTTCTACAAGCGAATCAACAAGGTGTTCTTCACCGAGTCTATCCCCAAGGCTCCCTCTGGCAAGATCCTGCGAAAGGACCTGCGAGCCAAGCTGGCTAACGGCCTGTAA |
| *VvVST1* | ATGGCCTCTGTGGAAGAGTTCCGAAACGCCCAGCGAGCCAAGGGACCCGCCACCATCCTGGCCATCGGCACCGCTACTCCCGACCACTGCGTGTACCAGTCTGACTACGCCGACTACTACTTCCGAGTGACCAAGTCTGAGCACATGACCGAGCTGAAGAAGAAGTTCAACCGAATCTGCGACAAGTCTATGATCAAGAAGCGGTACATCCACCTGACCGAGGAAATGCTCGAGGAACACCCCAACATCGGCGCCTACATGGCCCCTTCTCTGAACATCCGACAAGAGATCATCACCGCCGAGGTGCCCCGACTGGGCCGAGATGCCGCTCTGAAGGCCCTGAAGGAATGGGGACAGCCCAAGTCCAAGATCACCCACCTGGTGTTCTGCACCACCTCTGGCGTCGAGATGCCCGGTGCCGACTACAAGCTGGCCAACCTGCTGGGCCTCGAGACTTCTGTGCGACGAGTGATGCTGTACCACCAGGGCTGCTACGCTGGCGGCACCGTGCTGCGAACCGCCAAGGACCTGGCCGAGAACAACGCTGGCGCCCGAGTGCTGGTGGTGTGCTCTGAGATCACCGTGGTGACCTTCCGAGGTCCTTCTGAGGACGCCCTGGACTCTCTGGTCGGACAGGCCCTGTTCGGCGACGGATCTTCTGCCGTGATCGTGGGCTCTGACCCCGACGTGTCTATCGAGCGACCCCTGTTCCAGCTGGTGTCTGCTGCCCAGACCTTCATTCCCAACTCTGCCGGCGCTATCGCCGGAAACCTGCGAGAGGTGGGCCTGACCTTCCACCTGTGGCCTAACGTGCCCACTCTGATCTCTGAGAACATCGAGAAGTGTCTGACCCAGGCTTTCGACCCTCTGGGAATCTCTGACTGGAACTCTCTGTTCTGGATCGCTCACCCCGGTGGACCCGCTATCCTGGACGCCGTCGAGGCCAAGCTGAACCTGGAAAAGAAGAAGCTGGAAGCTACCCGACACGTGCTGTCTGAGTACGGCAACATGTCCTCTGCCTGCGTGCTGTTCATTCTGGACGAGATGCGAAAGAAGTCTCTGAAGGGCGAGAAGGCCACCACCGGCGAGGGACTCGACTGGGGAGTGCTGTTCGGCTTCGGACCCGGCCTGACCATCGAGACTGTGGTGCTGCACTCTGTGCCCACCGTGACCAACTAA |
| *EcEntC* | ATGGACACCTCTCTGGCCGAAGAGGTGCAGCAGACCATGGCCACTCTGGCTCCCAACCGATTCTTCTTCATGTCTCCCTACCGATCTTTCACCACCTCTGGCTGCTTCGCCCGATTCGACGAGCCCGCCGTGAACGGCGACTCTCCCGACTCGCCCTTCCAGCAGAAGCTGGCCGCTCTGTTCGCCGACGCCAAGGCTCAGGGCATCAAGAACCCCGTGATGGTGGGCGCTATCCCTTTCGACCCTCGACAGCCCTCTTCTCTGTACATCCCCGAGTCTTGGCAGTCTTTCTCGCGACAAGAGAAGCAGGCCTCTGCTCGACGATTCACCCGATCTCAGTCTCTGAACGTGGTCGAGCGACAGGCTATCCCCGAGCAGACCACCTTCGAGCAGATGGTGGCCCGAGCCGCCGCTCTGACCGCTACTCCCCAGGTGGACAAGGTGGTGCTGTCTCGACTGATCGACATCACCACCGACGCCGCCATCGACTCTGGCGTGCTGCTCGAGCGACTGATTGCTCAGAACCCTGTGTCTTACAACTTCCACGTGCCTCTCGCCGACGGCGGAGTGCTGCTGGGCGCTTCTCCCGAGCTGCTGCTGCGAAAGGACGGCGAGCGATTCTCTTCTATTCCCCTGGCCGGCTCTGCCCGACGACAGCCCGACGAGGTGCTGGACCGAGAGGCCGGCAACCGACTGCTGGCCTCTGAGAAGGACCGACACGAGCACGAGCTGGTGACCCAGGCCATGAAGGAAGTGCTGCGAGAGCGATCTTCTGAGCTGCACGTGCCCTCTTCGCCCCAGCTGATCACCACTCCTACTCTGTGGCACCTGGCTACCCCTTTTGAAGGCAAGGCCAACTCTCAAGAGAACGCCCTGACTCTCGCCTGCCTGCTGCATCCCACGCCTGCTCTGTCTGGCTTCCCTCACCAGGCCGCCACTCAGGTGATCGCCGAGCTGGAACCCTTCGACCGAGAGCTGTTCGGCGGCATCGTCGGCTGGTGCGACTCTGAAGGCAACGGCGAGTGGGTCGTGACCATCCGATGCGCCAAGCTCCGAGAGAACCAGGTGCGACTGTTCGCTGGCGCCGGAATCGTGCCCGCCTCGTCGCCCCTCGGAGAGTGGCGAGAGACTGGCGTGAAGCTGTCTACCATGCTGAACGTGTTCGGCCTGCACTAA |
| *PpPchB* | ATGAACTTCCCTCTGGTGGACCCCGACATGAAGACCCCTGAGCAGTGCTCTGGCCTGGACGACGTCCGATGCGGCATCGACGCCATGGACCAGCAGATCATTCAGGCCCTGGGCCGACGACTGGCCTACGTGAAGGCCGCTGCTCAGTTCAAGCCCACCGAGGACTCTATCGCTGCTCCCGAGCGAGTGGCCGCCATGCTGCCCCAGCGACGACAGTGGGCCGAGCAGGCCTCTCTGGACCCCATGTTCGTGGTGCCCCTGTTCGCCCAGATCATCCACTGGAACATTGCCCAGCAGGTCCGACACTGGCGACGGCAGCACGGCCTGGACCAGGGCGCCCAGGACGAGTAA |
| *PaAroZ* | ATGCCCTCTAAGCTGGCCATCACCTCTATGTCTCTGGGCCGATGCTACGCCGGCCACTCTTTCACCACCAAGCTGGACATGGCCCGAAAGTACGGCTACCAGGGCCTCGAGCTGTTCCACGAGGACCTGGCCGACGTGGCCTACCGACTGTCTGGCGAGACTCCCTCTCCTTGCGGCCCCTCGCCTGCCGCTCAGCTGTCTGCCGCTCGACAGATCCTGCGAATGTGCCAGGTGCGAAACATCGAGATCGTGTGCCTGCAGCCTTTCTCGCAGTACGACGGCCTGCTGGACCGAGAGGAACACGAGCGACGACTCGAGCAGCTGGAATTCTGGATCGAGCTGGCCCACGAGCTGGACACCGACATCATTCAGATCCCCGCCAACTTCCTGCCTGCCGAGGAAGTGACCGAGGACATCTCTCTGATCGTGTCTGACCTGCAAGAGGTGGCCGACATGGGACTGCAGGCTAACCCTCCTATCCGATTCGTGTACGAGGCTCTGTGCTGGTCTACCCGAGTGGACACCTGGGAGCGATCTTGGGAAGTCGTGCAGCGAGTGAACCGACCTAACTTCGGCGTGTGTCTGGACACCTTCAACATTGCCGGCCGAGTGTACGCTGACCCCACCGTGGCCTCTGGACGAACCCCTAACGCCGAAGAGGCCATCCGAAAGTCTATCGCCCGACTGGTCGAGCGAGTGGACGTGTCTAAGGTGTTCTACGTGCAGGTCGTGGACGCCGAGAAGCTGAAGAAGCCTCTGGTCCCCGGCCACCGATTCTACGACCCCGAGCAGCCCGCTCGAATGTCTTGGTCCCGAAACTGCCGACTGTTCTACGGCGAGAAGGACCGAGGCGCCTACCTGCCTGTGAAGGAAATCGCCTGGGCCTTCTTCAACGGCCTGGGCTTCGAAGGCTGGGTGTCTCTGGAACTGTTCAACCGACGAATGTCTGACACCGGCTTCGGAGTGCCCGAGGAACTCGCCCGACGAGGCGCCGTGTCTTGGGCCAAGCTGGTGCGAGACATGAAGATCACCGTGGACTCTCCCACTCAGCAGCAGGCTACCCAGCAGCCTATCCGAATGCTGTCTCTGTCTGCTGCCCTGTAA |
| *KpAroY.B* | ATGAAGCTGATCATCggtatgactggtgctacaggtgctccattgggtgttgctttgttgcaagcttTGCGAGACATGCCCGAGGTCGAGACTCACCTGGTGATGTCTAAGTGGGCCAAGACCACCATCGAGCTGGAAACCCCTTGGACCGCTCGAGAGGTGGCCGCTCTGGCCGACTTCTCTCACTCTCCCGCCGACCAGGCCGCTACCATCTCTTCTGGATCTTTCCGAACCGACGGAATGATCGTGATCCCCTGCTCTATGAAGACCCTGgctggtattagagctggttatgctgaaggtttggttggtagagctgctgatgttGTGCTGAAGGAAGGCCGAAAGCTGGTTCTGGTGCCCCGAGAGATGCCCCTGTCTACCATCCACCTCGAGAACATGCTGGCCCTGTCTCGAATGGGAGTCGCTATGGTGCCTCCTATGCCTGCCTACTACAACCATCCTGAGACTGTGGACGACATTACCAACCACATCGTGACCCGAGTGCTGGACCAGTTCGGCCTGGACTACCACAAGGCCCGACGATGGAACGGCCTGCGAACCGCCGAGCAGTTCGCCCAAGAGATCGAGTAA |
| *KpAroY.C^iso^* | ATGACCGCTCCTATCCAGGACCTGCGAGATGCTATCGCCCTGCTCCAGCAGCACGACAACCAGTACCTCGAGACTGACCATCCTGTGGACCCCAACGCCGAGCTGGCCGGCGTGTACCGACACATCGGAGCCGGCGGAACCGTGAAGCGACCCACTCGAATCGGACCCGCCATGATGTTCAACAACATCAAGGGCTACCCTCACTCTCGAATCCTGGTGGGCATGCACGCCTCTCGACAGCGAGCCGCTCTGCTGCTGGGCTGCGAGGCTTCTCAGCTGGCCCTCGAGGTCGGCAAGGCCGTGAAGAAGCCCGTCGCTCCCGTCGTGGTGCCCGCCTCTTCTGCTCCCTGCCAAGAGCAGATCTTCCTGGCTGACGACCCCGACTTCGACCTGCGAACCCTGCTGCCTGCTCACACCAACACTCCTATCGACGCTGGCCCCTTCTTCTGCCTGGGACTCGCCCTGGCTTCTGACCCCGTGGACGCCTCTCTGACCGACGTGACCATCCACCGACTGTGCGTGCAGGGCCGAGATGAGCTGTCTATGTTCCTGGCCGCTGGCCGACACATTGAGGTGTTCCGACAGAAGGCCGAGGCTGCCGGCAAGCCCCTGCCTATCACCATCAACATGGGACTCGACCCCGCCATCTACATCGGCGCCTGCTTCGAGGCTCCCACCACTCCTTTCGGCTACAACGAGCTGGGCGTCGCTGGCGCCCTGCGACAGCGACCCGTCGAGCTGGTCCAGGGCGTGTCTGTGCCCGAGAAGGCCATTGCTCGAGCCGAGATCGTGATTGAGGGCGAGCTGCTGCCCGGCGTGCGAGTGCGAGAGGACCAGCACACCAACTCTGGCCACGCCATGCCTGAGTTCCCCGGCTACTGTGGCGGCGCTAACCCCTCTCTGCCCGTGATCAAGGTGAAGGCCGTCACCATGCGAAACAACGCCATCCTGCAGACCCTGGTCGGACCCGGCGAGGAACACACTACCCTGGCCGGACTGCCCACCGAGGCCTCTATCTGGAACGCCGTCGAGGCCGCTATTCCCGGCTTCCTGCAGAACGTGTACGCCCACACCGCTGGCGGCGGAAAGTTCCTGGGAATCCTGCAGGTCAAGAAGCGACAGCCCGCCGACGAGGGCCGACAGGGCCAAGCTGCCCTGCTGGCCCTGGCCACCTACTCTGAGCTGAAGAACATCATTCTGGTGGACGAGGACGTGGACATCTTCGACTCTGACGACATTCTGTGGGCCATGACTACCCGAATGCAGGGCGACGTGTCTATTACCACCATTCCTGGCATCCGAGGCCACCAGCTGGACCCCTCTCAGACCCCTGAGTACTCGCCCTCTATCCGAGGCAACGGCATCTCTTGTAAGACCATTTTCGACTGCACCGTGCCTTGGGCTCTGAAGTCTCACTTCGAGCGAGCCCCTTTCGCCGACGTGGACCCTCGACCTTTCGCTCCCGAGTACTTCGCCCGACTCGAGAAGAACCAGGGCTCTGCCAAGTAA |
| *KpAroY.D* | ATGATCTGTCCCCGATGCGCCGACGAGAAGATCGAGGTGATGGCCACCTCTCCTGTGAAGGGCGTGTGGACCGTGTACCAGTGCCAGCACTGCCTGTACACCTGGCGAGACACCGAGCCTCTGCGACGAACCTCGCGAGAGCACTACCCCGAGGCCTTCCGAATGACCCAGAAGGACATCGACGAGGCTCCCCAGGTGCCTCACGTGCCTCCACTGCTGCCCGAGGACAAGCGATAA |
| *CaCatA* | ATGTCTCAGGCCTTCACCGAGTCTGTCAAGACCTCTCTGGGCCCCAACGCTACCCCTCGAGCCAAGAAGCTGATCGCCTCTCTGGTGCAGCACGTGCACGACTTCGCCCGAGAGAACCACCTGACCACCGAGGACTGGCTGTGGGGCGTCGACTTCATCAACCGAATCGGCCAGATGTCTGACTCTCGACGAAACGAGGGCATCCTGGTGTGCGACATCATCGGCCTCGAGACTCTGGTGGACGCCCTGACCAACGAGTCTGAGCAGTCTAACCACACCTCTTCCGCCATTCTGGGACCCTTCTACCTGCCTGACTCTCCTGTGTACCCCAACGGCGGCTCTATCGTGCAGAAGGCTATCCCCACCGACGTGAAGTGCTTCGTGCGAGGCAAGGTGACCGACACCGAGGGCAAGCCCCTCGGCGGAGCCCAGCTCGAGGTGTGGCAGTGCAACTCTGCCGGCTTCTACTCTCAGCAGGCCGACCACGACGGCCCCGAGTTCAACCTGCGAGGCACCTTCATCACCGACGACGAGGGAAACTACTCTTTCGAGTGTCTGCGACCTACTTCTTACCCCATTCCTTACGACGGACCCGCTGGCGACCTGCTGAAGATCATGGATCGACACCCCAACCGACCTTCTCACATCCACTGGCGAGTGTCTCACCCCGGCTACCACACTCTGATCACCCAGATCTACGACGCCGAGTGTCCCTACACCAACAACGACTCTGTGTACGCCGTGAAGGACGACATCATTGTGCACTTCGAGAAGGTGGACAACAAGGACAAGGACCTGGTCGGCAAGGTCGAGTACAAGCTGGACTACGACATCTCTCTGGCCACCGAGTCCTCTATCCAAGAGGCCCGAGCCGCCGCTAAGGCCCGACAGGACGCCGAGATCAAGCTGTAA |

**Supplementary information 3**

Specific yields for production assays (mg/L/OD_600_), degradation assays for *cis*,*cis*-muconic acid and salicylic acid, medium optimization experiments, effect on resveratrol titers of feedback-resistant *ARO4* and *ARO7*, and additional information on selection of point mutations in Aro7p.


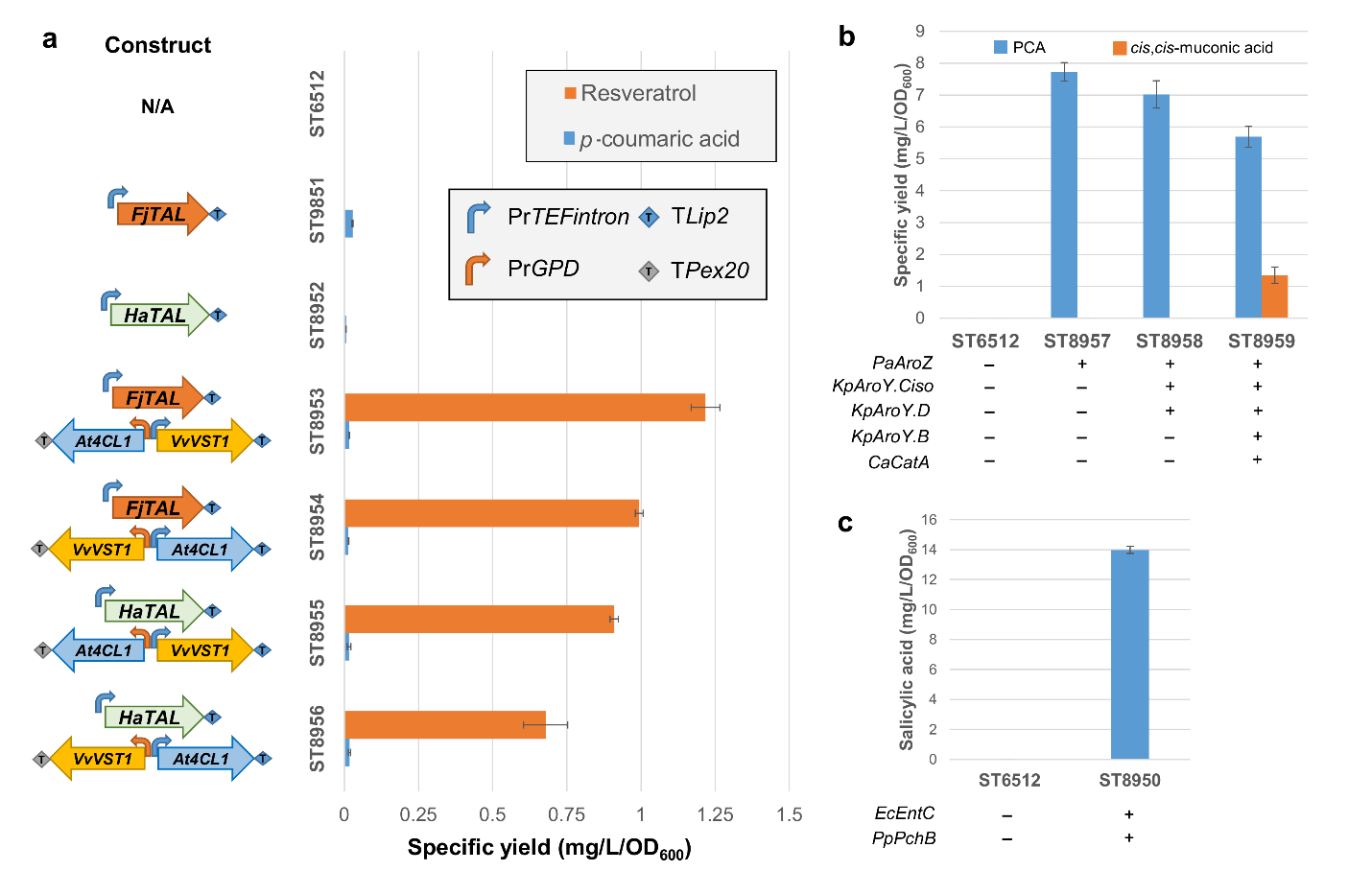


**Supplementary Figure S1. Specific yields of *p*-coumaric acid, resveratrol, *cis*,*cis*-muconic acid and salicylic in engineered *Y. lipolytica* strains**. Cultivations were carried out for 72 h in 24 deep-well plates containing mineral medium with 20 g/L glucose. Extracellular content was subjected to HPLC analysis. Error bars represent standard deviation from at least three biological replicates. “-“ and “+“ symbols indicate absence or presence of the corresponding genetic modification, respectively. A) Production of *p*-coumaric acid and resveratrol with supplementation of 2 mM L-tyrosine. B) Production of *cis*,*cis*-muconic acid and the pathway intermediate protocatechuic acid (PCA). C) Production of salicylic acid.


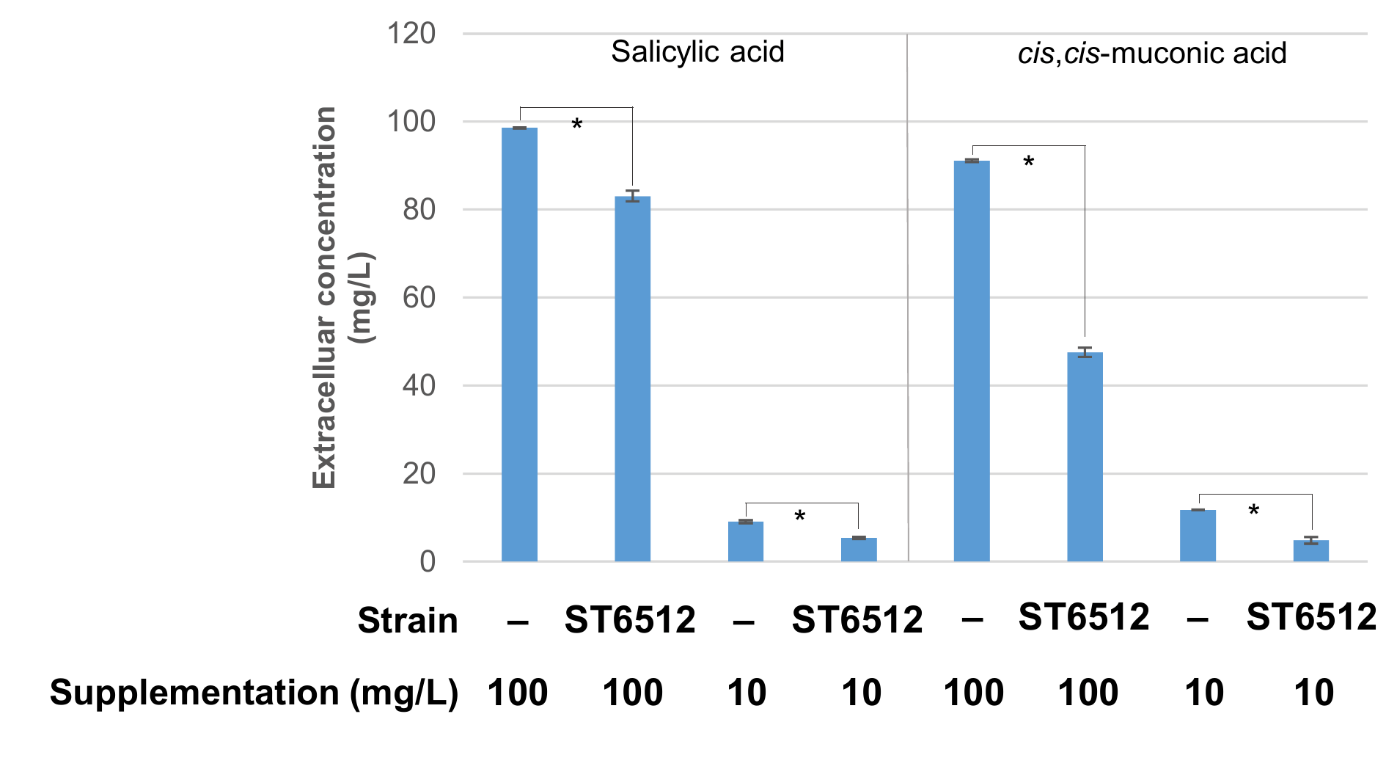


**Supplementary Figure S2. Degradation assays for *cis*,*cis*-muconic acid and salicylic acid in strain ST6512, at different supplementation concentrations**. “-“ represents a medium control, non-inoculated. Error bars represent standard deviation from at least three biological replicates. Statistical analysis was performed using Student's t-test (two-tailed; *P ≤ 0.05 two-sample unequal variance)


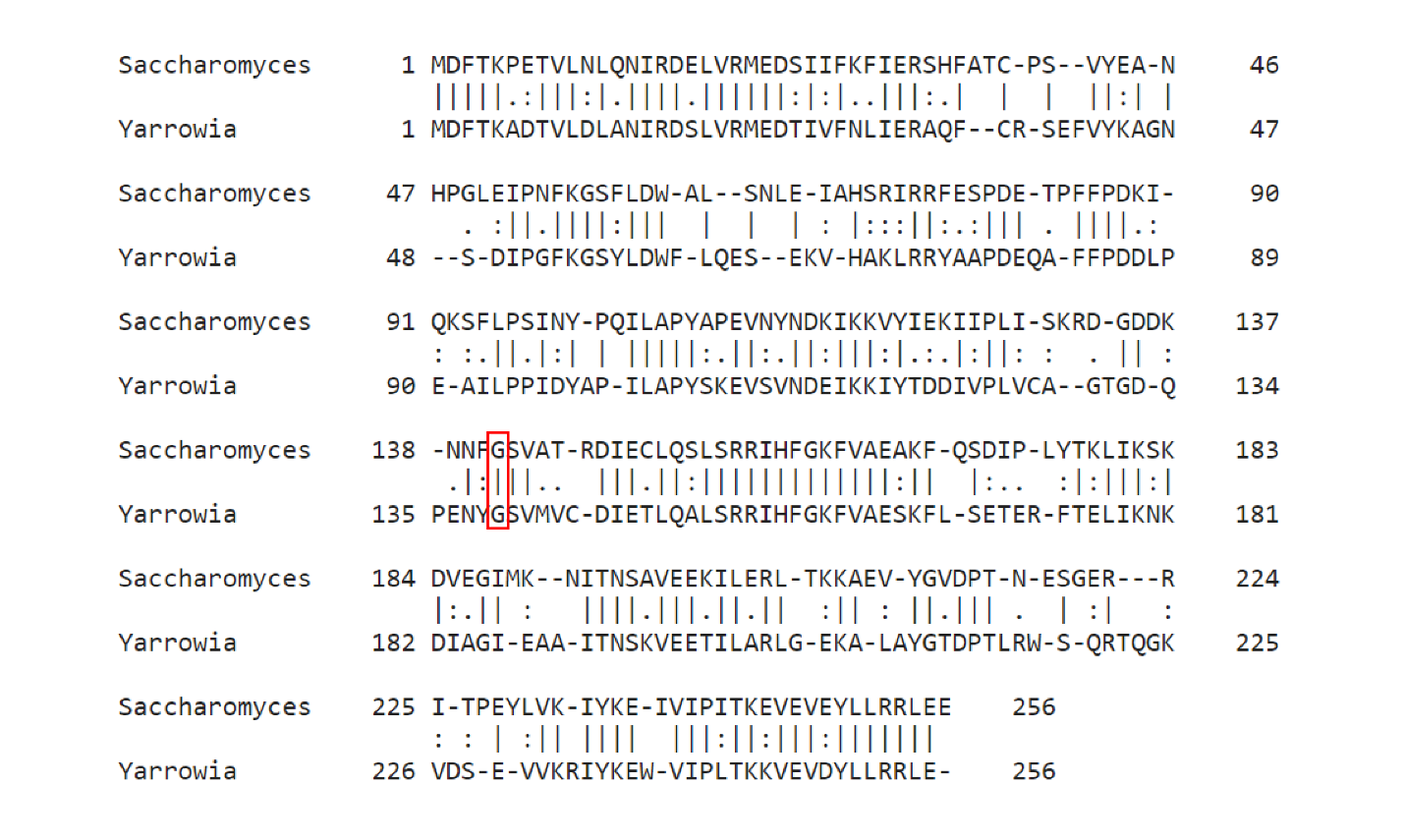


**Supplementary Figure S3. Pairwise alignment between *S. cerevisiae* and *Y. lipolytica* Aro7p**. Protein sequences were obtained from strains *S. cerevisiae* S288C (YPR060C) and *Y. lipolytica* CLIB89/W29 (YALI1_E20751p). In red, amino acid 141 that in *S. cerevisiae* confers feedback insensitivity when mutated from G to S (Luttik et al., 2008). Amino acid 139 in *Y. lipolytica* was subjected to the same replacement.


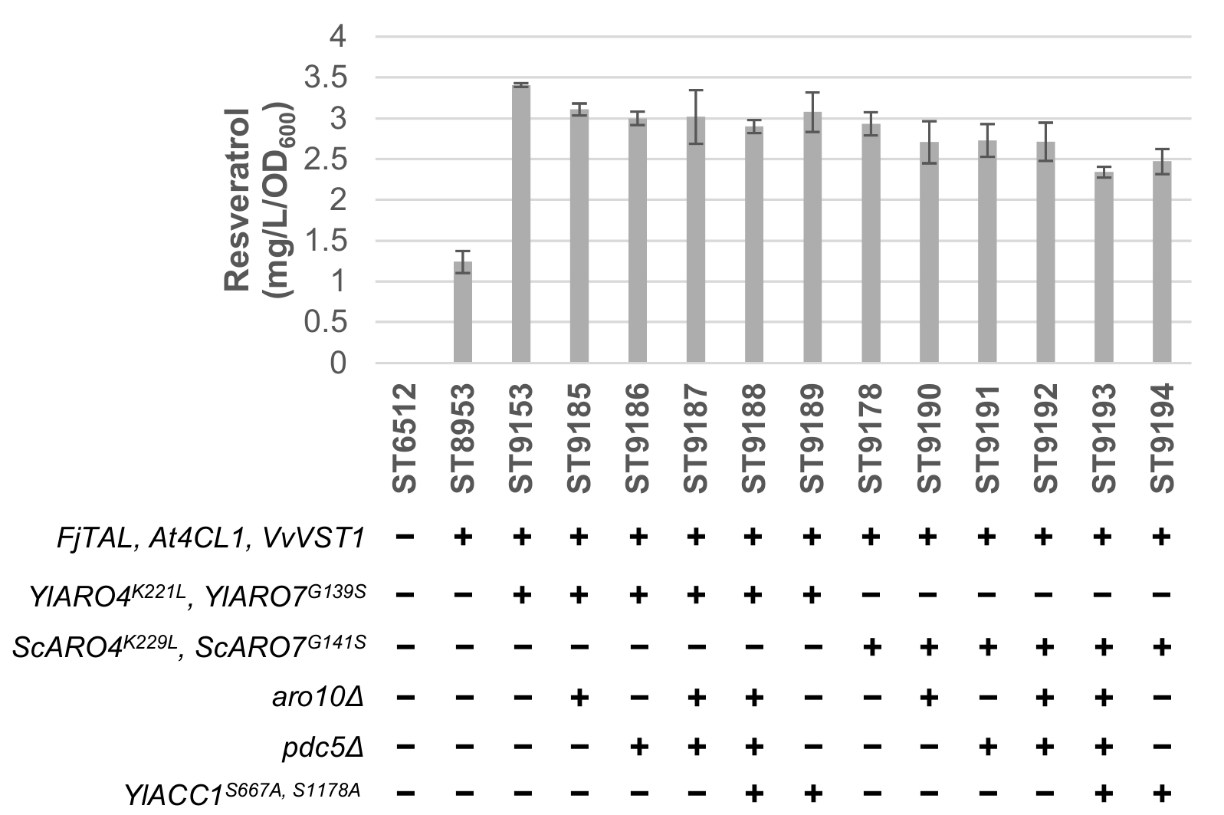


**Supplementary Figure S4. Specific resveratrol yield in engineered *Y. lipolytica* strains.** Cultivations were carried out for 72 h in 24 deep-well plates containing mineral medium with 20 g/L glucose. Extracellular content was subjected to HPLC analysis. “-“ and “+“ symbols indicate absence or presence of the corresponding genetic modification, respectively. Error bars represent standard deviation from at least three biological replicates. *p*-Coumaric acid was not detected in any of the strains.


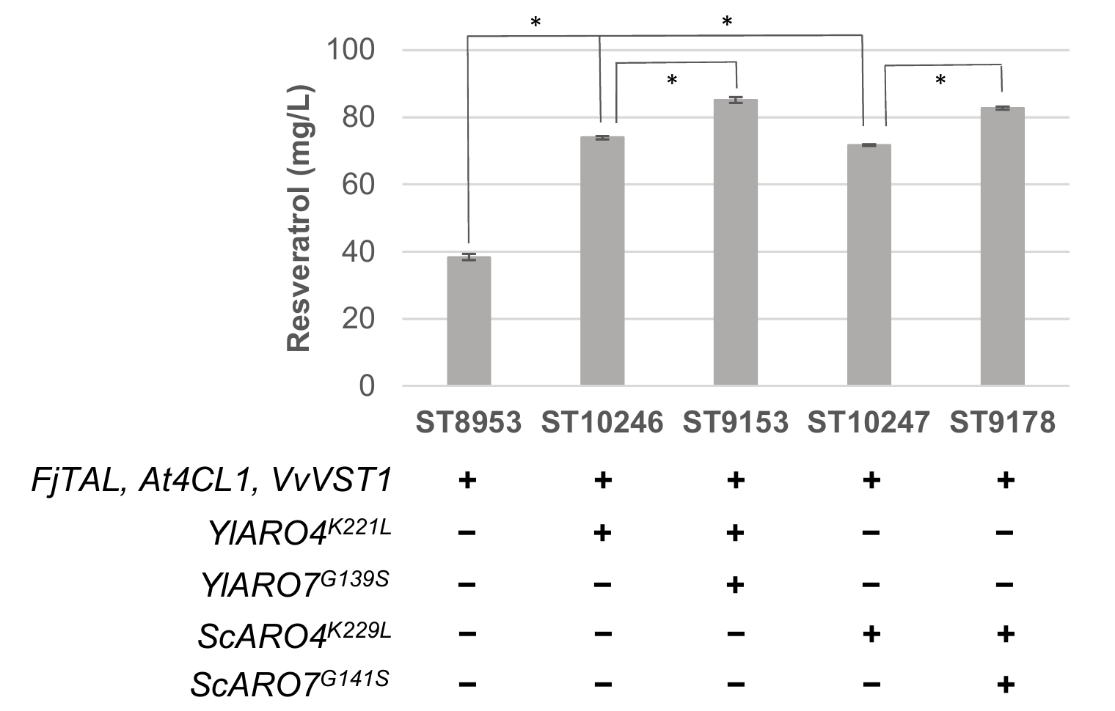


**Supplementary Figure S5. Resveratrol titer in engineered *Y. lipolytica* strains expressing feedback insensitive alleles of *ARO4* and *ARO7***. Cultivations were carried out for 72 h in 24 deep-well plates containing mineral medium with 20 g/L glucose. Extracellular content was subjected to HPLC analysis. “-“ and “+“ symbols indicate absence or presence of the corresponding genetic modification, respectively. Error bars represent standard deviation from at least three biological replicates. *p*-Coumaric acid was not detected in any of the strains. Statistical analysis was performed using Student's t-test (two-tailed; *P ≤ 0.001 two-sample unequal variance)


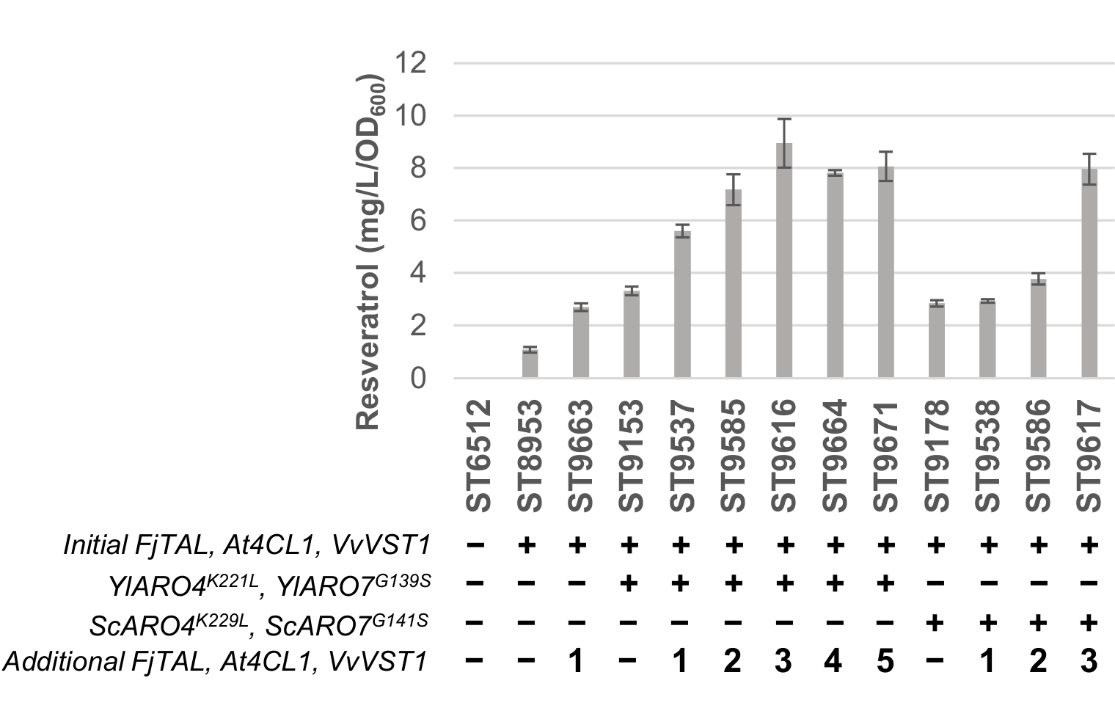


**Supplementary Figure S6. Effect on specific resveratrol yield upon integration of multiple copies of resveratrol biosynthetic genes**. Cultivations were carried out for 72 h in 24 deep-well plates containing mineral medium with 20 g/L glucose. Extracellular content was subjected to HPLC analysis. “-“ and “+“ symbols indicate absence or presence of the corresponding genetic modification, respectively. Digits show the number of additional copies of resveratrol biosynthetic genes integrated. Error bars represent standard deviation from at least three biological replicates. *p*-Coumaric acid was not detected in any of the strains.


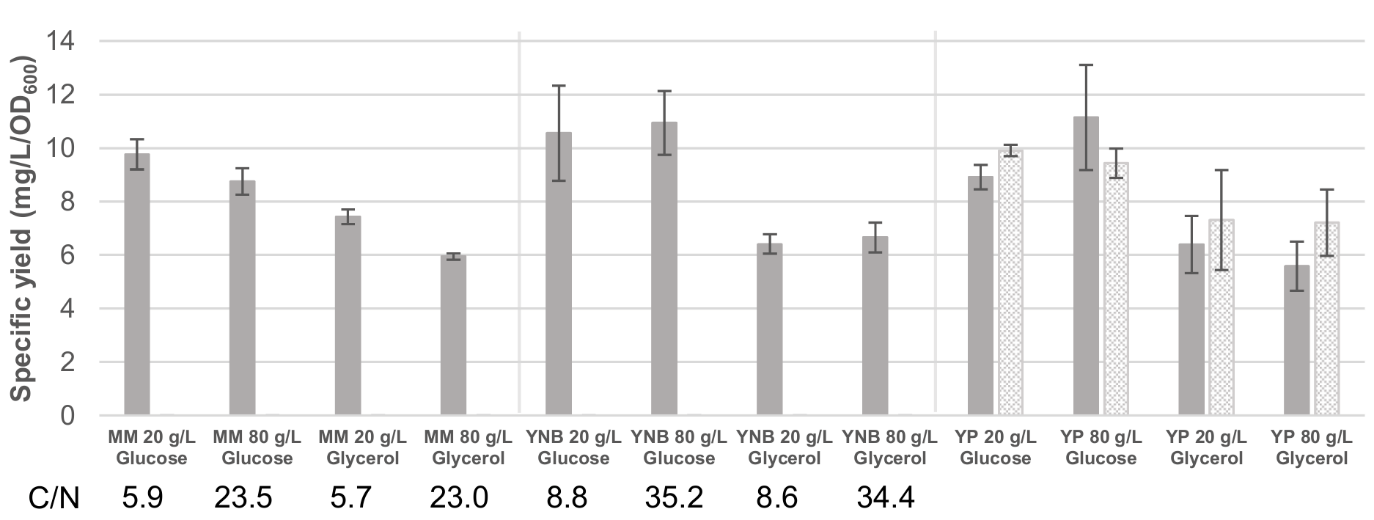


**Supplementary Figure S7. Specific resveratrol yield of ST9671 in different media.** Cultivations of strain ST9671 were carried out for 96 h in 24 deep-well plates using the media indicated for each condition. Extracellular content was subjected to HPLC analysis. Error bars represent standard deviation from at least three biological replicates. MM: mineral medium, YNB: Yeast Nitrogen Base without amino acids. YP: Yeast extract Peptone. C/N stands for the molar carbon/nitrogen ratio in defined media.



**Supplementary Figure S8. Cultivation profile of ST9671 in mineral medium (MM).** a) 20 g/L Glucose, b) 80 g/L glucose, c) 20 g/L glycerol, d) 80 g/L glycerol. Cultivations of strain ST9671 were carried out for 96 h in 24 deep-well plates and samples were taken every 24 h. Extracellular content was subjected to HPLC analysis. Error bars represent standard deviation from at least three biological replicates. No *p*-coumaric acid was detected.



**Supplementary Figure S9. Cultivation profile of ST9671 in Yeast Nitrogen Base without amino acids (YNB) medium.** a) 20 g/L Glucose, b) 80 g/L glucose, c) 20 g/L glycerol, d) 80 g/L glycerol. Cultivations of strain ST9671 were carried out for 96 h in 24 deep-well plates and samples were taken every 24 h. Extracellular content was subjected to HPLC analysis. Error bars represent standard deviation from at least three biological replicates. No *p*-coumaric acid was detected.



**Supplementary Figure S10. Cultivation profile of ST9671 in Yeast Peptone (YP) medium.** a) 20 g/L Glucose, b) 80 g/L glucose, c) 20 g/L glycerol, d) 80 g/L glycerol. Cultivations of strain ST9671 were carried out for 96 h in 24 deep-well plates and samples were taken every 24 h. Extracellular content was subjected to HPLC analysis. Error bars represent standard deviation from at least three biological replicates.

**Supplementary information 4**

**Information related to the fed-batch experiment carried out in bioreactors and analysis of intracellular and extracellular resveratrol content of strain ST9671 in small-scale cultivation.**

**Selection of initial feed rate and exponential increase was selected as follows:**

Exponential-feeding rate at time *t* follows the equation:

*F(t)* = *F_0_* . exp(*kt*)

with *k* being the exponential constant (h^-1^) and *F_0_* the initial feeding rate (g-feed/h).

To create a carbon-limited condition, *k* should be well below the maximum growth rate (*k*

<< *µ_max_*), and glucose should be fed below the rate of its consumption at any time:

*F(t) . C*_glc,feed_  < *r_glc_*(*t*), which also applies at the time of feeding start:

*F_0_ . C*_glc,feed_  *< r_glc_*,*_0_* ⇔ *F_0_ < r_glc_*,*_0_* / *C*_glc,feed_

Initial glucose consumption rate (*r_glc,0_*) can be determined by multiplying cell growth rate when feeding starts (*µ_0_*), the biomass yield on glucose (1/*Y_SX_*), and the amount of cells at the start of the feeding (*X_0_ . V_0_*):

*r_glc,0_* = *µ_0_ .* (1/*Y_SX_*) *. X_0_* . *V_0_*

The following assumptions were made based on measurements and available data for *Y. lipolytica*:

- *µ_0_* = 0.22 h^-1^ (growth rate at late exponential phase, measured)
- *Y_SX_* = 0.43 g-cell/g-glucose (Kerkhoven et al., 2016)
- *X_0_* = 10 g-cell/L (Marella et al., 2019)
- *V_0_* = 0.4 L (the volume of batch culture)

These values give *r_glc_* = 2.04 g-glucose/h. With glucose concentration in the feed, *C_glc,feed_* = 0.38 g-glucose/g-feed (calculated):

*F_0_ < r_glc,0_ / C_glc,feed_ =* 5.42 g-feed/h

- An *F_0_* of 1.0 g-feed/h was then selected to satisfy the above criteria.
- A *k* value of 0.05 h^-1^ was chosen to satisfy *k << µ_max_* criteria.

Eventually, the fed-batch feeding rate followed the following equation:

*F(t)* = 1.0 exp (0.05*t*)

F(t) was fixed when aeration & stirring rate reached maximum capacity.

For metrics calculations, the following parameters were considered:

Initial reactor volume: 400 mL

Feed medium added: 467.7 g = 383.3 mL

Volume extracted per sample: 3 mL

Base added: 68.7 ± 2.1 mL

Antifoam added: 27.6 mL


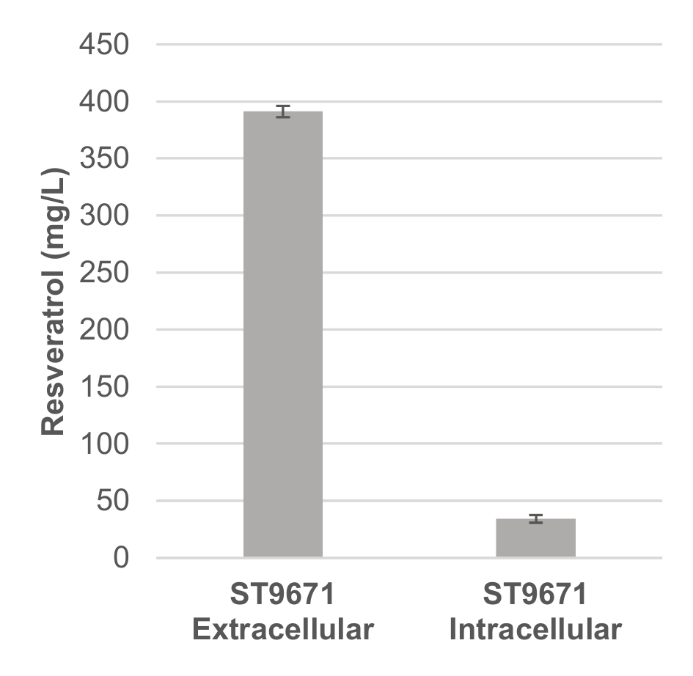


**Supplementary Figure S11. Analysis of extracellular and intracellular resveratrol concentrations in the top-producing strain ST9671**. Cultivations were carried out for 72 h in 24 deep-well plates containing mineral medium with 20 g/L glucose. Error bars represent standard deviation from at least three biological replicates. *p*-Coumaric acid was not detected in any of the strains.



**Supplementary Figure S12. Fed-batch fermentation in bioreactor of the highest producing strain ST9671.** Individual graphs for each of the reactors. a) Fermentation profile for reactor 1, b) Operational parameters for reactor 1, c) Fermentation profile for reactor 2, d) Operational parameters for reactor 2. Error bars for CDW (cell dry weight) represent standard deviation from two measurements of the same bioreactor. *p*-Coumaric acid was not detected. Black arrow represents the start of the feeding (22.35 h). Green arrows indicate antifoam 204 addition. Severe foaming was a constant issue throughout the cultivation. Manual addition of defoamer was carried out at the following times, as indicated by the green arrows: 22.42 h (0.1 mL), 37.02 h (2 mL), 38.40 h (3 mL), 48.11 h (0.5 mL), 62.73 h (1 mL), 64.10 h (3 mL), 71.21 h (4 mL), 72.70 h (1 mL), 75.62 h (2 mL), 76.50 h (5 mL), 79.37 h (2 mL), 82.11 (2 mL), 87.80 h (2 mL).

**
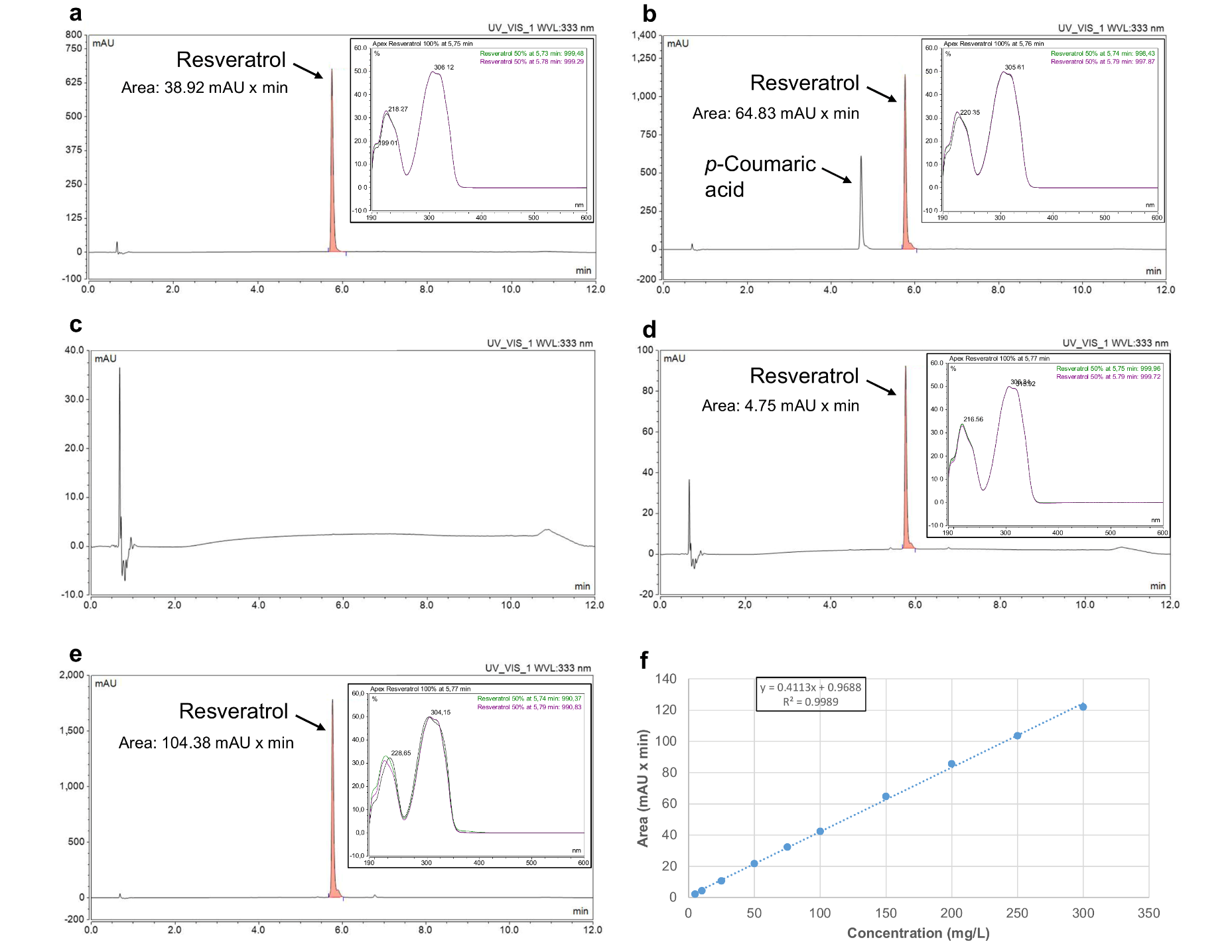
Supplementary Figure S13. HPLC analysis of fermentation samples for resveratrol quantification.** Chromatograms (absorbance at 333 nm) and resveratrol UV-Vis spectra of a) Resveratrol standard (90 mg/L), b) Resveratrol and *p*-coumaric acid standard (150 mg/L each), c) Reactor 1 sample (0 h, 1/50 dilution), d) Reactor 1 sample (33.20 h, 1/50 dilution), e) Reactor 1 sample (89.96 h, 1/50 dilution). f) External calibration curve used for resveratrol quantification in reactor samples. The UV-Vis spectra show the characteristic maximum absorbance at 306 nm of *trans*-resveratrol (Nour et al., 2012)


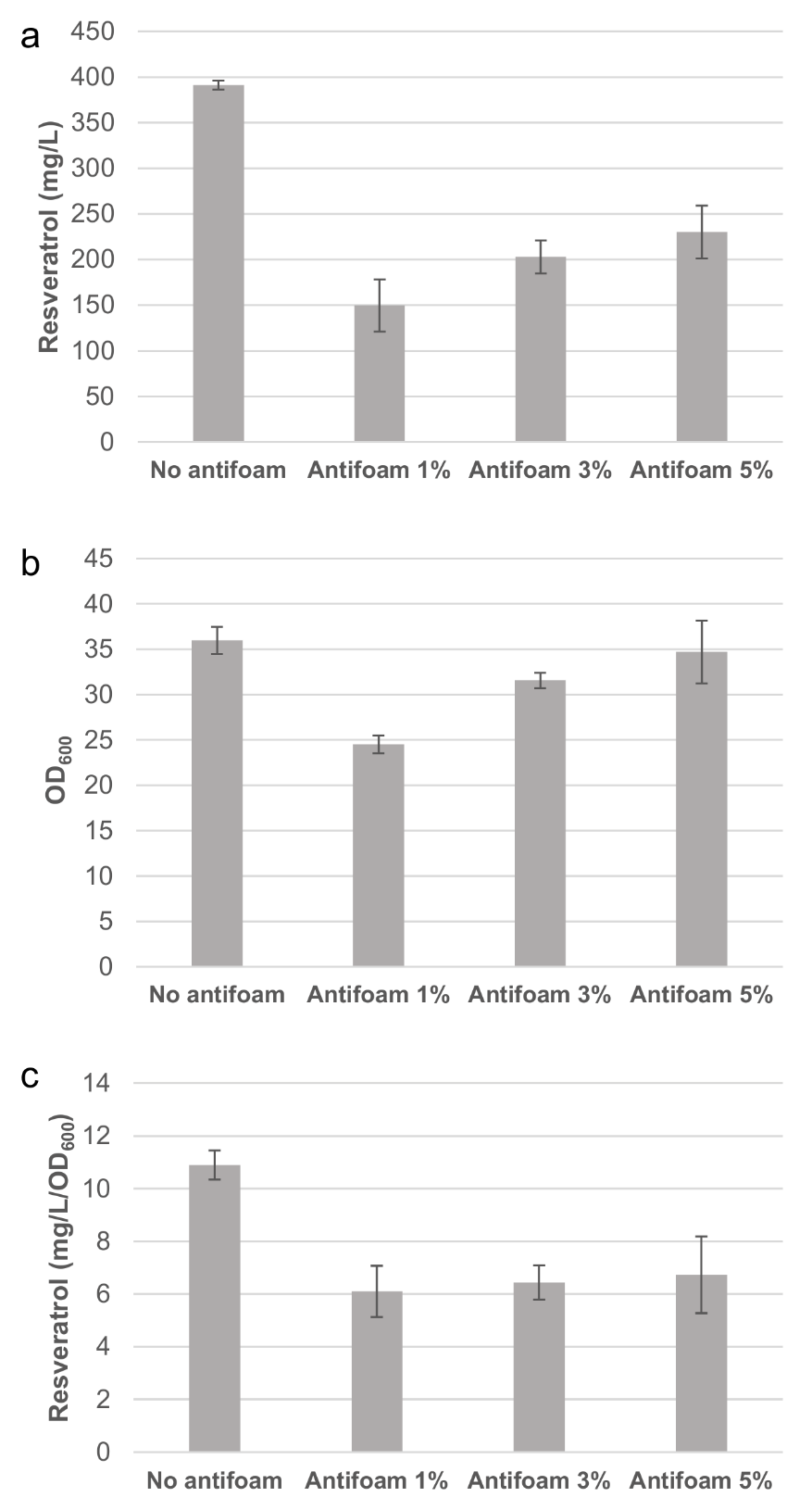


**Supplementary Figure S14. Antifoam effect on resveratrol titers in the top-producing strain ST9671.** Cultivations were carried out for 72 h in 24 deep-well plates containing mineral medium with 20 g/L glucose and the indicated v/v % of antifoam. Extracellular content was subjected to HPLC analysis. Error bars represent standard deviation from at least three biological replicates. a) Resveratrol titer. b) Optical density at 600 nm (OD_600_). c) Specific resveratrol yield.

**References:**

Afonso, M.S., Ferreira, S., Domingues, F.C., Silva, F., 2015. Resveratrol production in bioreactor: Assessment of cell physiological states and plasmid segregational stability. Biotechnology Reports 5, 7–13. https://doi.org/10.1016/j.btre.2014.10.008

Becker, J.V.W., Armstrong, G.O., van der Merwe, M.J., Lambrechts, M.G., Vivier, M.A., Pretorius, I.S., 2003. Metabolic engineering of *Saccharomyces cerevisiae* for the synthesis of the wine-related antioxidant resveratrol. FEMS Yeast Res 4, 79–85. https://doi.org/10.1016/S1567-1356(03)00157-0

Beekwilder, J., Wolswinkel, R., Jonker, H., Hall, R., Vos, C.H.R. de, Bovy, A., 2006. Production of Resveratrol in Recombinant Microorganisms. Appl. Environ. Microbiol. 72, 5670–5672. https://doi.org/10.1128/AEM.00609-06

Bhan, N., Xu, P., Khalidi, O., Koffas, M.A.G., 2013. Redirecting carbon flux into malonyl-CoA to improve resveratrol titers: Proof of concept for genetic interventions predicted by OptForce computational framework. Chemical Engineering Science, Synthetic Biology 103, 109–114. https://doi.org/10.1016/j.ces.2012.10.009

Braga, A., Oliveira, J., Silva, R., Ferreira, P., Rocha, I., Kallscheuer, N., Marienhagen, J., Faria, N., 2018. Impact of the cultivation strategy on resveratrol production from glucose in engineered *Corynebacterium glutamicum*. Journal of Biotechnology 265, 70–75. https://doi.org/10.1016/j.jbiotec.2017.11.006

Camacho-Zaragoza, J.M., Hernández-Chávez, G., Moreno-Avitia, F., Ramírez-Iñiguez, R., Martínez, A., Bolívar, F., Gosset, G., 2016. Engineering of a microbial coculture of *Escherichia coli* strains for the biosynthesis of resveratrol. Microbial Cell Factories 15, 163. https://doi.org/10.1186/s12934-016-0562-z

Choi, O., Wu, C.-Z., Kang, S.Y., Ahn, J.S., Uhm, T.-B., Hong, Y.-S., 2011. Biosynthesis of plant-specific phenylpropanoids by construction of an artificial biosynthetic pathway in *Escherichia coli*. J Ind Microbiol Biotechnol 38, 1657–1665. https://doi.org/10.1007/s10295-011-0954-3

Gaspar, P., Dudnik, A., Neves, A.R., Förster, J., 2016. Engineering *Lactococcus lactis* for stilbene production. Presented at the 28th International Conference on Polyphenols.

Gu, Y., Ma, J., Zhu, Y., Ding, X., Xu, P., 2020. Engineering *Yarrowia lipolytica* as a Chassis for *De Novo* Synthesis of Five Aromatic-Derived Natural Products and Chemicals. ACS Synth. Biol. https://doi.org/10.1021/acssynbio.0c00185

Holkenbrink, C., Dam, M.I., Kildegaard, K.R., Beder, J., Dahlin, J., Belda, D.D., Borodina, I., 2018. EasyCloneYALI: CRISPR/Cas9-Based Synthetic Toolbox for Engineering of the Yeast *Yarrowia lipolytica*. Biotechnology Journal 13, 1700543. https://doi.org/10.1002/biot.201700543

Huang, L.L., Xue, Z., Zhu, Q.Q., 2006. Method for the production of resveratrol in a recombinant oleaginous microorganism. WO2006125000A2.

Kallscheuer, N., Vogt, M., Stenzel, A., Gätgens, J., Bott, M., Marienhagen, J., 2016. Construction of a *Corynebacterium glutamicum* platform strain for the production of stilbenes and (2S)-flavanones. Metabolic Engineering 38, 47–55. https://doi.org/10.1016/j.ymben.2016.06.003

Kang, S.-Y., Lee, J.K., Choi, O., Kim, C.Y., Jang, J.-H., Hwang, B.Y., Hong, Y.-S., 2014. Biosynthesis of methylated resveratrol analogs through the construction of an artificial biosynthetic pathway in *E. coli*. BMC Biotechnology 14, 67. https://doi.org/10.1186/1472-6750-14-67

Katsuyama, Y., Funa, N., Horinouchi, S., 2007a. Precursor-directed biosynthesis of stilbene methyl ethers in *Escherichia coli*. Biotechnol. J. 2, 1286–1293. https://doi.org/10.1002/biot.200700098

Katsuyama, Y., Funa, N., Miyahisa, I., Horinouchi, S., 2007b. Synthesis of Unnatural Flavonoids and Stilbenes by Exploiting the Plant Biosynthetic Pathway in *Escherichia coli*. Chemistry & Biology 14, 613–621. https://doi.org/10.1016/j.chembiol.2007.05.004

Kerkhoven, E.J., Pomraning, K.R., Baker, S.E., Nielsen, J., 2016. Regulation of amino-acid metabolism controls flux to lipid accumulation in *Yarrowia lipolytica*. npj Systems Biology and Applications 2, 16005. https://doi.org/10.1038/npjsba.2016.5

Li, M., Kildegaard, K.R., Chen, Y., Rodriguez, A., Borodina, I., Nielsen, J., 2015. *De novo* production of resveratrol from glucose or ethanol by engineered *Saccharomyces cerevisiae*. Metabolic Engineering 32, 1–11. https://doi.org/10.1016/j.ymben.2015.08.007

Li, M., Schneider, K., Kristensen, M., Borodina, I., Nielsen, J., 2016. Engineering yeast for high-level production of stilbenoid antioxidants. Scientific Reports 6, 36827. https://doi.org/10.1038/srep36827

Lim, C.G., Fowler, Z.L., Hueller, T., Schaffer, S., Koffas, M.A.G., 2011. High-Yield Resveratrol Production in Engineered *Escherichia coli*. Appl. Environ. Microbiol. 77, 3451–3460. https://doi.org/10.1128/AEM.02186-10

Liu, X., Lin, J., Hu, H., Zhou, B., Zhu, B., 2016. *De novo* biosynthesis of resveratrol by site-specific integration of heterologous genes in *Escherichia coli*. FEMS Microbiol Lett 363. https://doi.org/10.1093/femsle/fnw061

Luttik, M.A.H., Vuralhan, Z., Suir, E., Braus, G.H., Pronk, J.T., Daran, J.M., 2008. Alleviation of feedback inhibition in *Saccharomyces cerevisiae* aromatic amino acid biosynthesis: Quantification of metabolic impact. Metabolic Engineering 10, 141–153. https://doi.org/10.1016/j.ymben.2008.02.002

Marella, E.R., Dahlin, J., Dam, M.I., ter Horst, J., Christensen, H.B., Sudarsan, S., Wang, G., Holkenbrink, C., Borodina, I., 2019. A single-host fermentation process for the production of flavor lactones from non-hydroxylated fatty acids. Metabolic Engineering. https://doi.org/10.1016/j.ymben.2019.08.009

Nour, V., Trandafir, I., Muntean, C., 2012. Ultraviolet Irradiation of *Trans*-Resveratrol and HPLC Determination of *Trans*-Resveratrol and *Cis*-Resveratrol in Romanian Red Wines. J Chromatogr Sci 50, 920–927. https://doi.org/10.1093/chromsci/bms091

Palmer, C.M., Miller, K.K., Nguyen, A., Alper, H.S., 2020. Engineering 4-coumaroyl-CoA derived polyketide production in *Yarrowia lipolytica* through a β-oxidation mediated strategy. Metabolic Engineering 57, 174–181. https://doi.org/10.1016/j.ymben.2019.11.006

Park, S.R., Yoon, J.A., Paik, J.H., Park, J.W., Jung, W.S., Ban, Y.-H., Kim, E.J., Yoo, Y.J., Han, A.R., Yoon, Y.J., 2009. Engineering of plant-specific phenylpropanoids biosynthesis in *Streptomyces venezuelae*. Journal of Biotechnology 141, 181–188. https://doi.org/10.1016/j.jbiotec.2009.03.013

Rodriguez, A., Kildegaard, K.R., Li, M., Borodina, I., Nielsen, J., 2015. Establishment of a yeast platform strain for production of *p*-coumaric acid through metabolic engineering of aromatic amino acid biosynthesis. Metabolic Engineering 31, 181–188. https://doi.org/10.1016/j.ymben.2015.08.003

Shin, S.-Y., Han, N.S., Park, Y.-C., Kim, M.-D., Seo, J.-H., 2011. Production of resveratrol from *p*-coumaric acid in recombinant *Saccharomyces cerevisiae* expressing 4-coumarate:coenzyme A ligase and stilbene synthase genes. Enzyme and Microbial Technology 48, 48–53. https://doi.org/10.1016/j.enzmictec.2010.09.004

Shin, S.-Y., Jung, S.-M., Kim, M.-D., Han, N.S., Seo, J.-H., 2012. Production of resveratrol from tyrosine in metabolically engineered *Saccharomyces cerevisiae*. Enzyme and Microbial Technology 51, 211–216. https://doi.org/10.1016/j.enzmictec.2012.06.005

Sydor, T., Schaffer, S., Boles, E., 2010. Considerable Increase in Resveratrol Production by Recombinant Industrial Yeast Strains with Use of Rich Medium. Appl. Environ. Microbiol. 76, 3361–3363. https://doi.org/10.1128/AEM.02796-09

Trantas, E., Panopoulos, N., Ververidis, F., 2009. Metabolic engineering of the complete pathway leading to heterologous biosynthesis of various flavonoids and stilbenoids in *Saccharomyces cerevisiae*. Metabolic Engineering 11, 355–366. https://doi.org/10.1016/j.ymben.2009.07.004

Wang, S., Zhang, S., Xiao, A., Rasmussen, M., Skidmore, C., Zhan, J., 2015. Metabolic engineering of *Escherichia coli* for the biosynthesis of various phenylpropanoid derivatives. Metabolic Engineering 29, 153–159. https://doi.org/10.1016/j.ymben.2015.03.011

Wang, Y., Halls, C., Zhang, J., Matsuno, M., Zhang, Y., Yu, O., 2011. Stepwise increase of resveratrol biosynthesis in yeast *Saccharomyces cerevisiae* by metabolic engineering. Metabolic Engineering 13, 455–463. https://doi.org/10.1016/j.ymben.2011.04.005

Watts, K.T., Lee, P.C., Schmidt-Dannert, C., 2006. Biosynthesis of plant-specific stilbene polyketides in metabolically engineered *Escherichia coli*. BMC Biotechnology 6, 22. https://doi.org/10.1186/1472-6750-6-22

Wu, J., Liu, P., Fan, Y., Bao, H., Du, G., Zhou, J., Chen, J., 2013. Multivariate modular metabolic engineering of *Escherichia coli* to produce resveratrol from l-tyrosine. Journal of Biotechnology 167, 404–411. https://doi.org/10.1016/j.jbiotec.2013.07.030

Wu, J., Zhou, P., Zhang, X., Dong, M., 2017. Efficient *de novo* synthesis of resveratrol by metabolically engineered *Escherichia coli*. J Ind Microbiol Biotechnol 44, 1083–1095. https://doi.org/10.1007/s10295-017-1937-9

Yang, Y., Lin, Y., Li, L., Linhardt, R.J., Yan, Y., 2015. Regulating malonyl-CoA metabolism via synthetic antisense RNAs for enhanced biosynthesis of natural products. Metabolic Engineering 29, 217–226. https://doi.org/10.1016/j.ymben.2015.03.018

Zhang, E., Guo, X., Meng, Z., Wang, J., Sun, J., Yao, X., Xun, H., 2015. Construction, expression, and characterization of *Arabidopsis thaliana* 4CL and *Arachis hypogaea* RS fusion gene 4CL::RS in *Escherichia coli*. World J Microbiol Biotechnol 31, 1379–1385. https://doi.org/10.1007/s11274-015-1889-z

Zhang, Y., Li, S.-Z., Li, J., Pan, X., Cahoon, R.E., Jaworski, J.G., Wang, X., Jez, J.M., Chen, F., Yu, O., 2006. Using Unnatural Protein Fusions to Engineer Resveratrol Biosynthesis in Yeast and Mammalian Cells. J. Am. Chem. Soc. 128, 13030–13031. https://doi.org/10.1021/ja0622094
